# Prediction and associations of preterm birth and its subtypes with eicosanoid enzymatic pathways and inflammatory markers

**DOI:** 10.1038/s41598-019-53448-z

**Published:** 2019-11-19

**Authors:** Max T. Aung, Youfei Yu, Kelly K. Ferguson, David E. Cantonwine, Lixia Zeng, Thomas F. McElrath, Subramaniam Pennathur, Bhramar Mukherjee, John D. Meeker

**Affiliations:** 1 Department of Environmental Health Sciences, University of Michigan School of Public Health, Ann Arbor, MI USA; 2 Department of Biostatistics, University of Michigan School of Public Health, Ann Arbor, MI USA; 3 Epidemiology Branch, National Institute of Environmental Health Sciences, Research Triangle Park, Durham, NC USA; 4 Division of Maternal and Fetal Medicine, Brigham and Women’s Hospital, Harvard Medical School, Boston, MA USA; 5 Department of Internal Medicine-Nephrology, University of Michigan, Ann Arbor, MI USA; 6 Michigan Regional Comprehensive Metabolomics Resource Core, University of Michigan, Ann Arbor, MI USA; 7 Department of Molecular and Integrative Physiology, University of Michigan, Ann Arbor, MI USA; 8 Department of Epidemiology, University of Michigan School of Public Health, Ann Arbor, MI USA

**Keywords:** Predictive markers, Epidemiology, Risk factors

## Abstract

Endogenous signaling molecules derived from lipids, peptides, and DNA, are important regulators of physiological processes during pregnancy. The effect of their collective impact on preterm birth (delivery < 37 weeks gestation) is understudied. We aimed to characterize the associations and predictive capacity of an extensive panel of eicosanoids, immune biomarkers, oxidative stress markers, and growth factors towards preterm birth and its subtypes. We conducted a cross-sectional study of pregnant women (recruited < 15 weeks gestation) in the LIFECODES birth cohort, which included 58 cases of preterm birth and 115 controls that delivered term. Among the cases there were 31 cases who had a spontaneous preterm birth (cases who had spontaneous preterm labor and/or preterm premature rupture of membranes) and 25 that had preterm birth associated with aberrant placentation (cases who had preeclampsia and/or intrauterine growth restriction) and 2 cases that could not be sufficiently categorized as either. We analyzed single biomarker associations with each preterm birth outcome using multiple logistic regression. Adaptive elastic-net was implemented to perform a penalized multiple logistic regression on all biomarkers simultaneously to identify the most predictive biomarkers. We then organized biomarkers into biological groups and by enzymatic pathways and applied adaptive elastic-net and random forest to evaluate the accuracy of each group for predicting preterm birth cases. The majority of associations we observed were for spontaneous preterm birth, and adaptive elastic-net identified 5-oxoeicosatetraenoic acid, resolvin D1, 5,6-epoxy-eicsatrienoic acid, and 15-deoxy-12,14-prostaglandin J2 as most predictive. Overall, lipid biomarkers performed the best at separating cases from controls compared to other biomarker categories (adaptive elastic-net AUC = 0.78 [0.62, 0.94], random forest AUC = 0.84 [0.72, 0.96]). Among the enzymatic pathways that differentiate eicosanoid metabolites, we observed the highest prediction of overall preterm birth by lipoxygenase metabolites using random forest (AUC = 0.83 [0.69, 0.96]), followed by cytochrome p450 metabolites using adaptive elastic-net (AUC = 0.74 [0.52, 0.96]). In this study we translate biological hypothesis into the language of modern machine learning. Many lipid biomarkers were highly associated with overall and spontaneous preterm birth. Among eicosanoids, lipoxygenase and cytochrome p450 products performed best in identifying overall and spontaneous preterm birth. The combination of lipid biomarkers may have good utility in clinical settings to predict preterm birth.

## Introduction

Preterm birth is a significant public health issue that occurs in approximately 10% of live births in the U.S., and remains the leading cause of neonatal mortality^1,2^. Babies who are born preterm have increased risk for health conditions later in life, including neurodevelopmental and sensory disorders, physical disabilities, cardiovascular disease, and impaired respiratory function^3,4^. Live births are broadly classified as preterm if they occurred at less than 37 weeks gestation, and there is great heterogeneity in clinical presentation and other circumstances contributing to preterm birth. Therefore, differentiation of preterm birth cases by clinical subtypes provides greater insight on the underlying antecedent mechanisms of preterm delivery. For example, spontaneous preterm births can involve spontaneous preterm labor or premature rupture of membranes, which is determined by the presence of vaginal pooling and either documentation of nitrazine-positive testing or ferning followed by regular uterine activity^5^. Whereas preterm birth associated with aberrant placentation is dominated by the presentation of preeclampsia (encompassing onset of hypertension and proteinuria) or intrauterine growth restriction^5^.

During pregnancy, systemic oxidative stress – the imbalance of reactive oxygen species and antioxidant molecules – can lead to inflammation and perturbation of several physiological processes that are integral for the progression of a healthy pregnancy. For example, oxidative stress and inflammation can cause tissue damage and alter vascularization, both of which can be harmful during critical stages of pregnancy, such as placentation, quiescence, cervical ripening, and induction of labor^6^. Furthermore, several biomarkers of oxidative stress and inflammation serve as signaling molecules that are integral for not only regulating maternal hormones but also facilitating cross talk between the maternal circulation and fetal compartment^7,8^.

We have previously reported associations between preterm birth and select biomarkers of inflammation and oxidative stress^9,10^. For inflammation, this included the general marker C-reactive protein (CRP) as well as a panel of cytokines; for oxidative stress, this included 8-isoprostane, a marker of lipid peroxidation, and 8-hydroxydeoxyguanosine, a marker of oxidative DNA damage. These represent only a small fraction of the repertoire of signaling molecules that regulate inflammation during pregnancy. Nonetheless, we learned that the pro-inflammatory cytokine interleukin-6 was associated with increased risk for spontaneous preterm birth, while the anti-inflammatory cytokine interleukin-10 was elevated among cases of preterm birth associated with aberrant placentation^9^. Among oxidative stress markers, we found that overall and spontaneous preterm birth were positively associated with 8-isoprostane and inversely associated with 8-hydroxydeoxyguanosine^10^.

We sought to expand our panel of biomarkers to investigate the combined effects of those previously measured biomarkers in addition to a newly measured panel of eicosanoids – a large class of lipid metabolites. Eicosanoids are metabolized from large fatty acids such as arachidonic acid and linoleic acid and produced through many enzymatic pathways, including three broad families of enzymes: lipoxygenases (LOX), cytochrome P450 (CYP450), and cyclooxygenases (COX)^11^. Eicosanoids are intimately connected to immunological signaling molecules and inflammation, and are therefore potent regulators of physiological processes^11^. Animal studies also indicate overlapping consequences clustered by enzyme specific metabolites. For example, metabolites produced through LOX pathways resulted in inflammation, vascular remodeling, and altered renal function^12^. Furthermore, although many eicosanoids, such as prostaglandins, can cause pro-inflammatory stimulation of target tissues, some groups of eicosanoids such as resolvins can be anti-inflammatory and resolve inflammation^13,14^. Altogether, the effects of eicosanoids on inflammation and cardiovascular activity provide a mechanistic link for preterm birth^15^.

Our primary study aim was to evaluate a group of 65 biomarkers in association with preterm birth and its subtypes. By measuring a large panel of eicosanoids and other biomarkers of inflammation and oxidative stress, we obtain greater coverage of signaling molecules, which could potentially increase the capacity to predict preterm birth. However, there are also major challenges that arise from analyzing high dimensional data, including multicollinearity and the risk for false discoveries due to multiple testing. We integrated variable selection and machine learning methods to circumvent some of the challenges with high-dimensional data. Our secondary study aim was to focus more deeply on the biological context of observed associations by grouping our analyses based on categories of molecules, and then by enzymatic pathways within the eicosanoids panel. We hypothesized that the combination of biomarkers would predict preterm birth, and that select pathways among eicosanoids will have differential predictive capacity. We translated this hypothesis by using a grouped variable selection framework.

## Results

The demographic characteristics of the subjects in this study are summarized in Table 1 according to their case and control status. In general, the controls had lower BMI, higher education level and were predominantly white subjects compared to the three types of cases, however these differences were not statistically significant. Patients with spontaneous preterm had lower BMI than those with preterm birth associated with aberrant placentation. The majority of patients were privately insured for both cases and controls.

**Table 1:** Characteristics of all participants in the subset sample from the LIFECODES prospective birth cohort (n = 173).

	All Preterm (n = 58)	Spontaneous Preterm (n = 31)	Preterm associated with aberrant placentation (n = 25)	Other Preterm (n = 2)	Controls (n = 115)
Timing of sample collection (gestational weeks)^a^	25.7 (1.3) [23.7, 28.4]	25.8 (1.2) [24.0, 28.4]	25.5 (1.4) [23.7, 28.4]	25.5 (1.5) [24.4, 26.6]	26.2 (1.2) [23.1, 28.9]
Duration between sample collection and delivery (gestational weeks)^a^	8.6 (3.3) [1.0, 13.0]	8.1 (3.4) [1.3, 12.9]	9.1 (3.2) [1.0, 13.0]	10.1 (2.8) [8.1, 12.1]	12.9 (1.6) [8.7, 16.9]
Age^a^	32.3 (5.0) [20.9, 46.4]	31.3 (4.6) [20.9, 40.1]	33.7 (5.5) [25.8, 46.4]	31.0 (1.6) [29.8, 32.1]	32.9 (4.3) [21.4, 43.5]
BMI at Initial Visit^b^					
Normal	22 (38%)	14 (45%)	7 (28%)	1 (50%)	62 (54%)
Overweight	15 (26%)	9 (29%)	5 (20%)	1 (50%)	34 (29.5%)
Obesity	21 (36%)	8 (26%)	13 (52%)	—	19 (16.5%)
Race^b^					
White	32 (55%)	14 (45.2%)	17 (68%)	1 (50%)	76 (66%)
African American	8 (14%)	2 (6.4%)	6 (24%)	—	11 (10%)
Other	18 (31%)	15 (48.4%)	2 (8%)	1 (50%)	28 (24%)
Education Level^b^					
High school degree	12 (21%)	6 (19.3%)	6 (24%)	—	12 (10%)
Technical school	9 (16%)	6 (19.3%)	3 (12%)	—	11 (10%)
Junior college or some college	17 (29%)	8 (26%)	9 (36%)	—	40 (35%)
College graduate	20 (34%)	11 (35.4%)	7 (28%)	2 (100%)	52 (45%)
Health Insurance Provider^b^					
Private/HMO/Self-pay	48 (84%)	25 (81%)	21 (88%)	2 (100%)	105 (91%)
Medicaid/SSI/MassHealth	9 (16%)	6 (19%)	3 (12%)	—	10 (9%)

^a^Continuous variables presented as: mean (standard deviation) [min, max].

^b^Categorical variables presented as: count (percent).

### Association of preterm birth with single biomarkers

**Figure 1:**
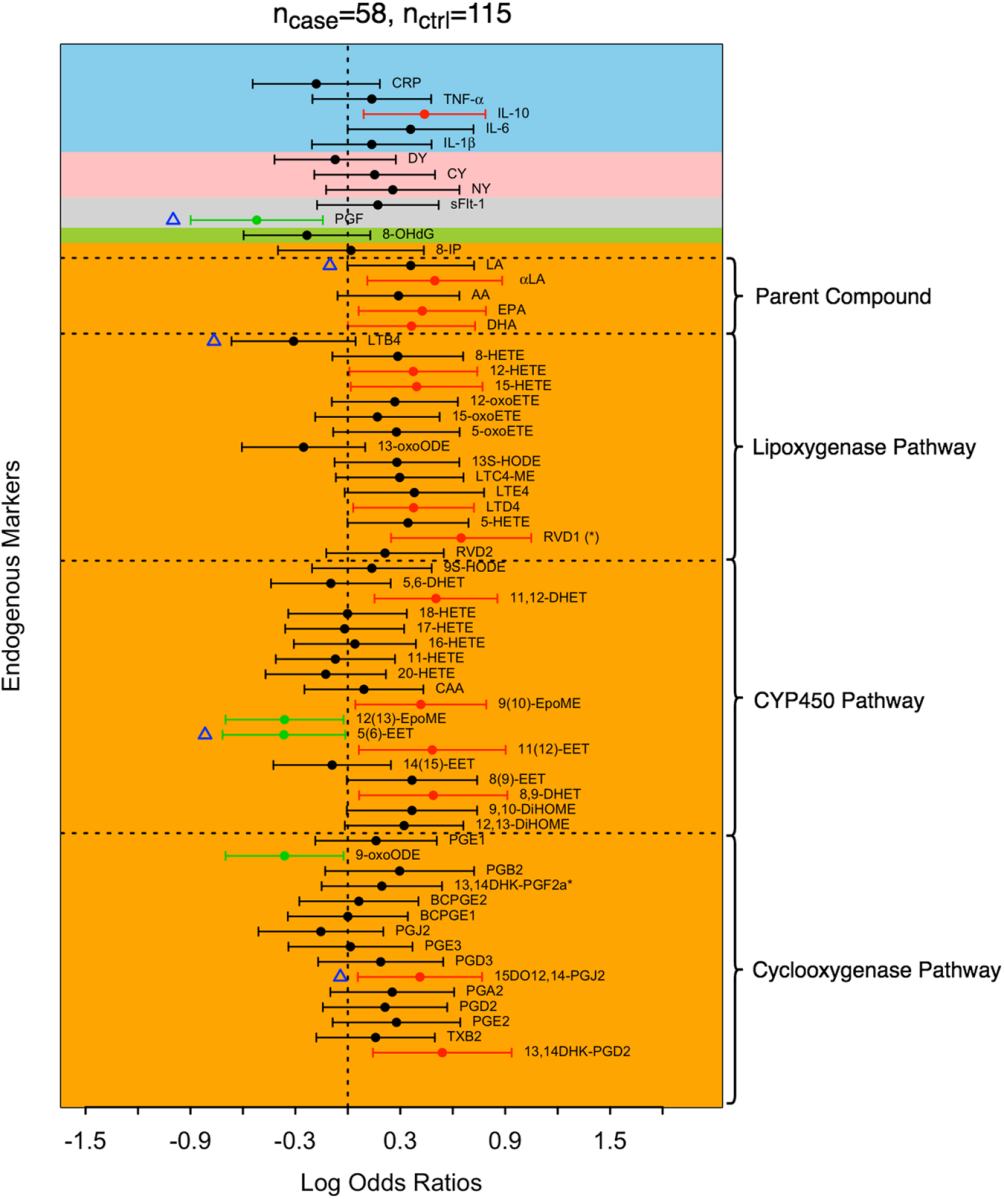
Log odds ratios of all preterm birth. Positive and negative log odds ratios with p < 0.05 are represented by red and green dots, respectively. Biomarkers that remain significant after controlling the false discovery rate at 0.1 are labeled by (*) in their names. Biomarkers selected by adaptive elastic-net applied to all biomarkers are labeled by blue triangles. The blocks are shaded according to their corresponding major groups: lipid damage markers (orange), DNA damage marker (green), angiogenic factors (grey), protein damage marker (pink), and inflammatory markers (blue).

**Figure 2:**
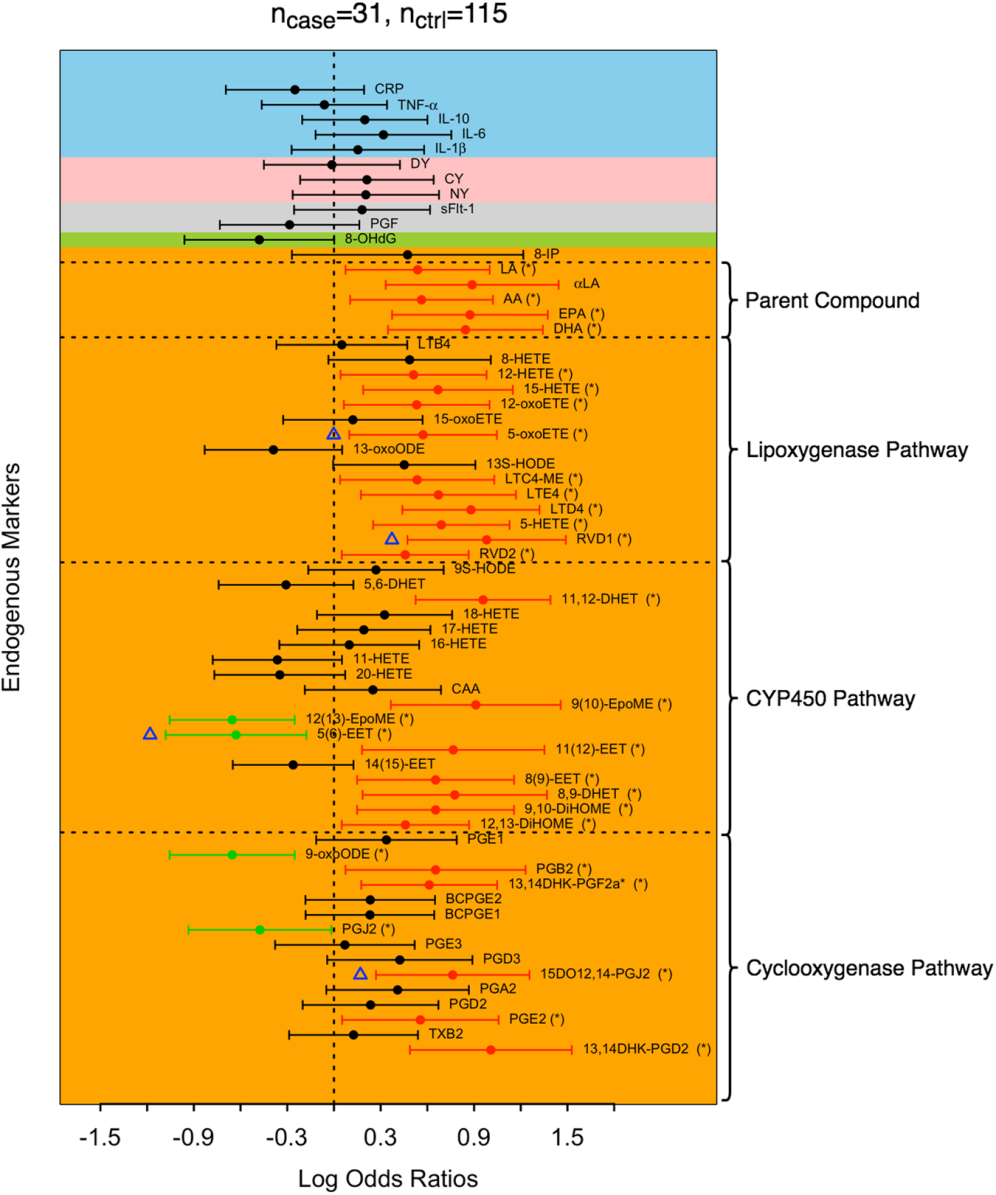
Log odds ratios of spontaneous preterm birth. Positive and negative log odds ratios with p < 0.05 are represented by red and green dots, respectively. Biomarkers that remain significant after controlling the false discovery rate at 0.1 are labeled by (*) in their names. Biomarkers selected by adaptive elastic-net applied to all biomarkers are labeled by blue triangles. The blocks are shaded according to their corresponding major groups: lipid damage markers (orange), DNA damage marker (green), angiogenic factors (grey), protein damage marker (pink), and inflammatory markers (blue).

**Figure 3:**
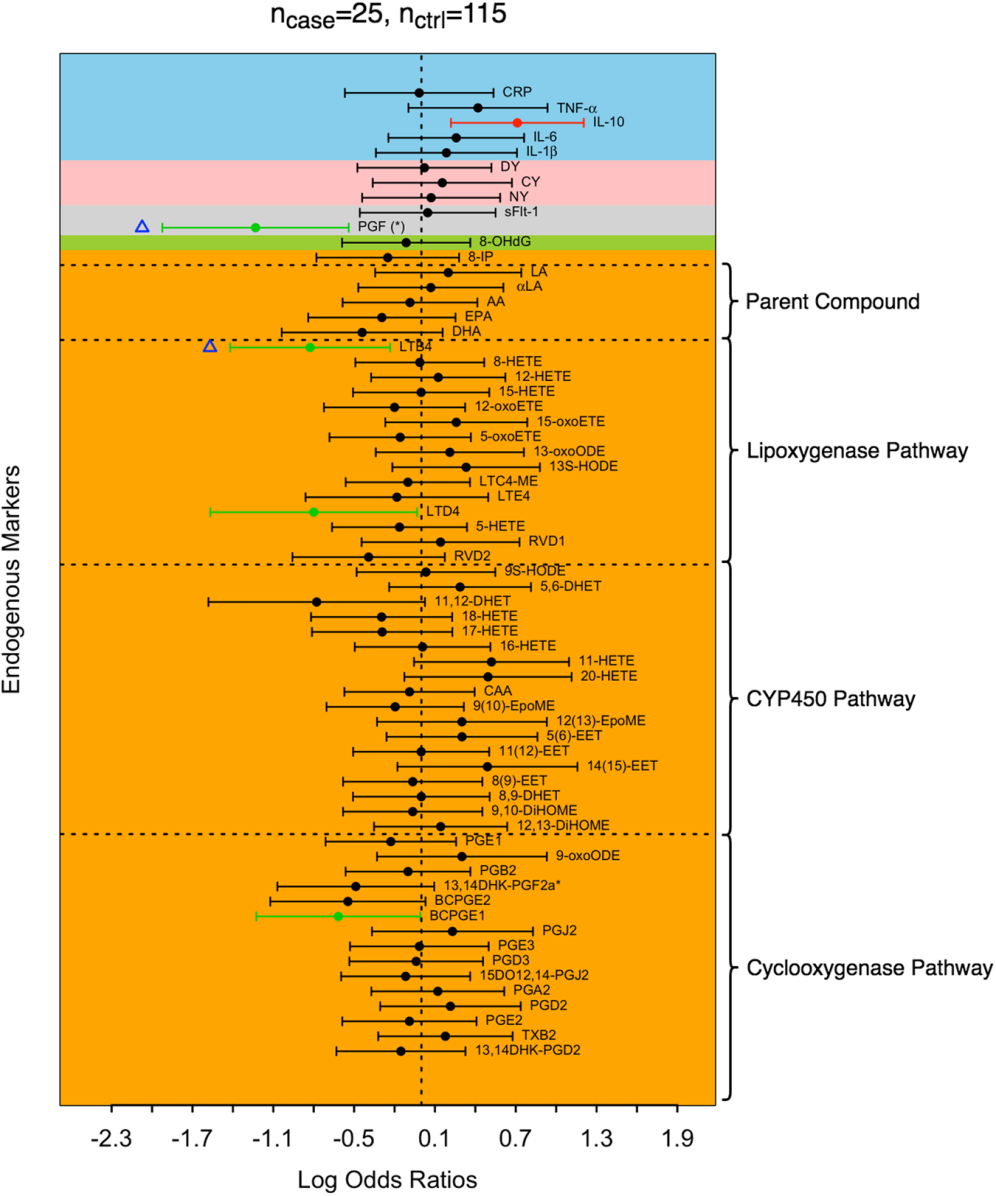
Log odds ratios of preterm birth associated with aberrant placentation. Positive and negative log odds ratios with p < 0.05 are represented by red and green dots, respectively. Biomarkers that remain significant after controlling the false discovery rate at 0.1 are labeled by (*) in their names. Biomarkers selected by adaptive elastic-net applied to all biomarkers are labeled by blue triangles. The blocks are shaded according to their corresponding major groups: lipid damage markers (orange), DNA damage marker (green), angiogenic factors (grey), protein damage marker (pink), and inflammatory markers (blue).

Figures 1–3 respectively present the estimates of log odds ratios of all preterm birth, spontaneous preterm birth, and preterm birth associated with aberrant placentation along with their 95% confidence interval for each biomarker. In particular, 14 biomarkers (13,14-dihydro-15-keto-prostaglandin [13,14DHK-PGD2], 15-deoxy-12,14-prostaglandin J2 [15DO12,14-PGJ2], 8,9-dihydroxy-eicosatrienoic acid [8,9-DHET], 11,12-epoxy-eicosatrienoic acid [11(12)-EET], 9,10-epoxy-octadecenoic acid [9(10)-EpoME], 11,12-dihydroxy-eicosatrienoic acid [11,12-DHET], resolvin D1 [RVD1], leukotriene D4 [LTD4], 15-hydroxy-eicosatetraenoic acid [15-HETE], 12-hydroxy-eicosatetraenoic acid [12-HETE], docosahexaenoic acid [DHA], eicosapentaenoic acid [EPA], α-linolenic acid [αLA], and interleukin-10 [IL-10]) were significantly associated with increased odds of preterm birth of any type at the level of 0.05. The lipid biomarkers that were associated with preterm birth are involved in all enzymatic pathways: the COX pathway (13,14DHK-PGD2, 15DO12,14-PGJ2), CYP450 pathway (8,9-DHET, 11(12)-EET, 9(10)-EpoME, 11,12-DHET), LOX pathway (RVD1, LTD4, 15-HETE, 12-HETE), or belong to the parent compound group (DHA, EPA, αLA). Four markers (9-oxooctadeca-dienoic acid [9-oxoODE], 12,13-epoxy-octadecenoic acid [12(13)-EpoME], 5,6-epoxy-eicsatrienoic acid [5(6)-EET], and placental growth factor [PGF]) had a significant protective effect on overall preterm birth. After controlling the false discovery rate (FDR) at 0.1, only RVD1 remained significant (Fig. 1).

Combined with the biomarkers that were associated with overall preterm birth, we observed fourteen additional lipid biomarkers associated with increased risk for spontaneous preterm birth (Fig. 2). These biomarkers included prostaglandin E2 [PGE2], 13,14-dihydro-15-keto-prostaglandin F2α [13,14DHK-PGF2α*], prostaglandin B2 [PGB2], 12,13-dihydroxy-octadecenoic acid [12,13-DiHOME], 9,10-dihydroxy-octadecenoic acid [9,10-DiHOME], 8,9-epoxy-eicosatrienoic acid [8(9)-EET], resolvin D2 [RVD2], 5- hydroxy-eicosatetraenoic acid [5-HETE], leukotriene E4 [LTE4], leukotriene C4 methyl-ester [LTC4-ME], 5-oxoeicosatetraenoic acid [5-oxoETE], 12-oxoeicosatetraenoic acid [12-oxoETE], arachidonic acid [AA], and linoleic acid [LA]. Prostaglandin J2 [PGJ2] was inversely associated with odds of spontaneous preterm birth but not with overall preterm birth. None of the non-lipid markers had significant associations with spontaneous preterm birth. With an FDR of 0.1, all of the biomarkers that were significantly associated with spontaneous preterm birth, regardless of the direction of associations, remained significant (Fig. 2). We observed fewer biomarkers in association with preterm birth associated with aberrant placentation compared to spontaneous preterm birth, with IL-10 being associated with a significantly increased odds ratio, and bicyclo-prostaglandin E1 [BCPGE1], LTD4, leukotriene B4 [LTB4], and PGF having protective associations (Fig. 3). The FDR corrected results revealed that PGF is the only biomarker that was significantly linked to preterm birth associated with aberrant placentation after the Benjamini-Hochberg procedure. Due to the sparsity of associations between the set of biomarkers and preterm birth associated with aberrant placentation, we focused our subsequent analysis on overall and spontaneous preterm birth. The specific values of log odds ratios and their 95% confidence intervals for overall preterm birth and subtypes are reported in Supplemental Table 1.

### Association of preterm birth with groups of biomarkers

**Figure 4:**
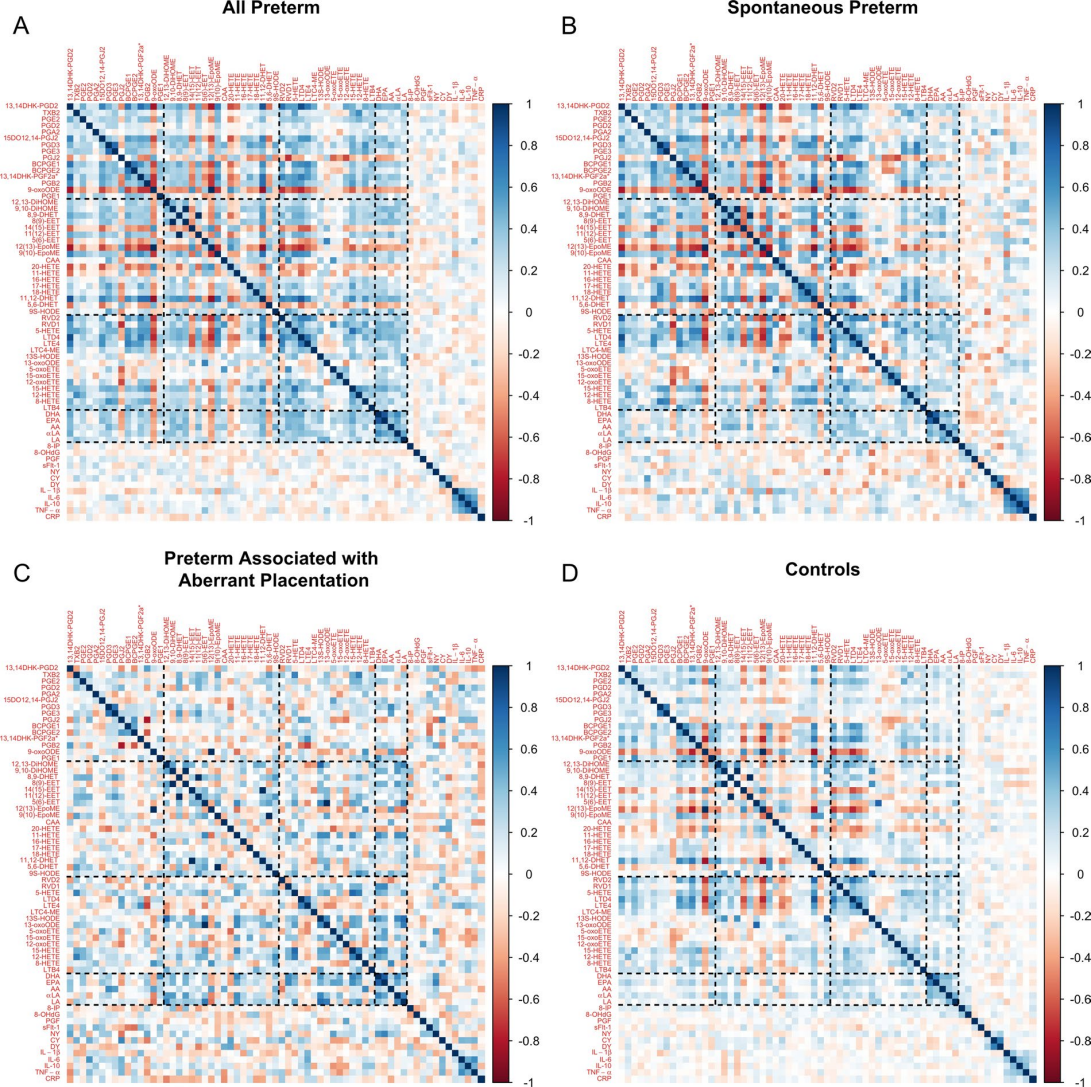
Correlations among biomarkers in cases and controls. Correlations were calculated among all preterm birth (**A**), spontaneous preterm birth (**B**), placental preterm birth (**C**), and controls (**D**). Eicosanoids were grouped by biological pathways (dashed lines).

We observed moderate to strong correlations between biomarkers (Fig. 4). Correlations among biomarkers were in general stronger in preterm birth cases, and spontaneous preterm birth in particular, compared to controls. For example, for spontaneous preterm, the median positive and negative pairwise correlations of biomarkers in the COX pathway were 0.33 and −0.33 (Fig. 4B), respectively, while their counterparts for the controls were 0.21 and −0.16, respectively (Fig. 4D). The correlations of the combined samples weighted for case-control sampling (Supplemental Fig. 2) were close to those of controls (Fig. 4D). Five biomarkers (15DO12,14-PGJ2, 5(6)-EET, LTB4, LA, and PGF) were identified by adaptive elastic-net as having a non-null effect on preterm birth overall (Fig. 1), the first four of which were lipid damage markers and the last one was an angiogenic factor. When we restricted the outcome to spontaneous preterm birth, 15DO12,14-PGJ2, 5(6)-EET, RVD1 and 5-oxoETE were associated with spontaneous preterm birth according to the results of adaptive elastic-net (Fig. 2). For preterm birth associated with aberrant placentation, only PGF and LTB4 were selected (Fig. 3). The corresponding adaptive elastic-net point estimates of the log odds ratios are reported in Supplemental Table 1.

**Figure 5:**
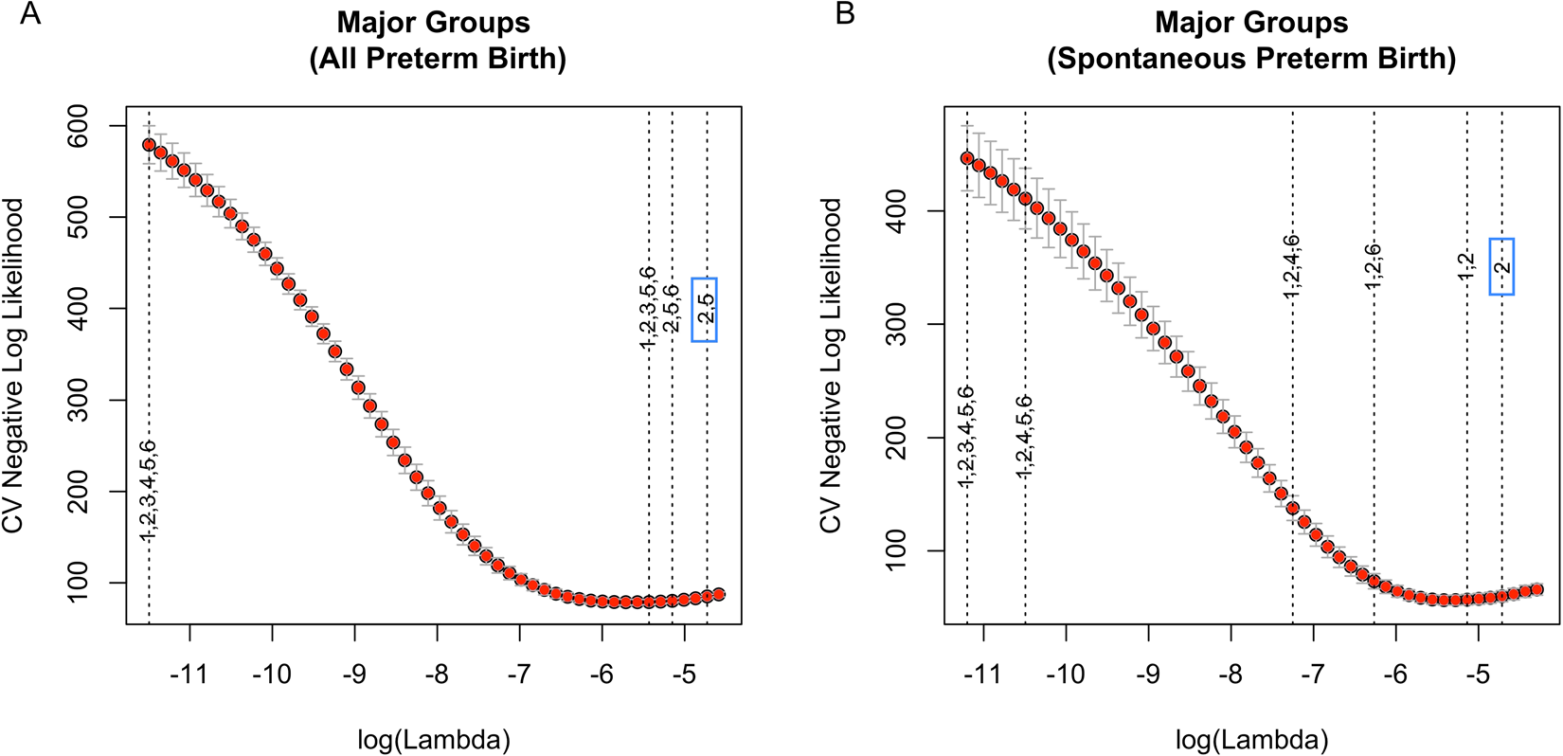
Sparse-group lasso on the five major groups for (**A**) all preterm birth and (**B**) spontaneous preterm birth. The dashed lines indicate the value of log(λ) corresponding to the first time the whole group of variables are excluded from the model as log(λ) increases. The numbers 1 to 6 on the dashed lines indicate covariates (1), **lipid damage markers** (2), DNA damage marker (3), angiogenic factors (4), **protein damage markers** (5), and inflammatory markers (6), respectively.

**Figure 6:**
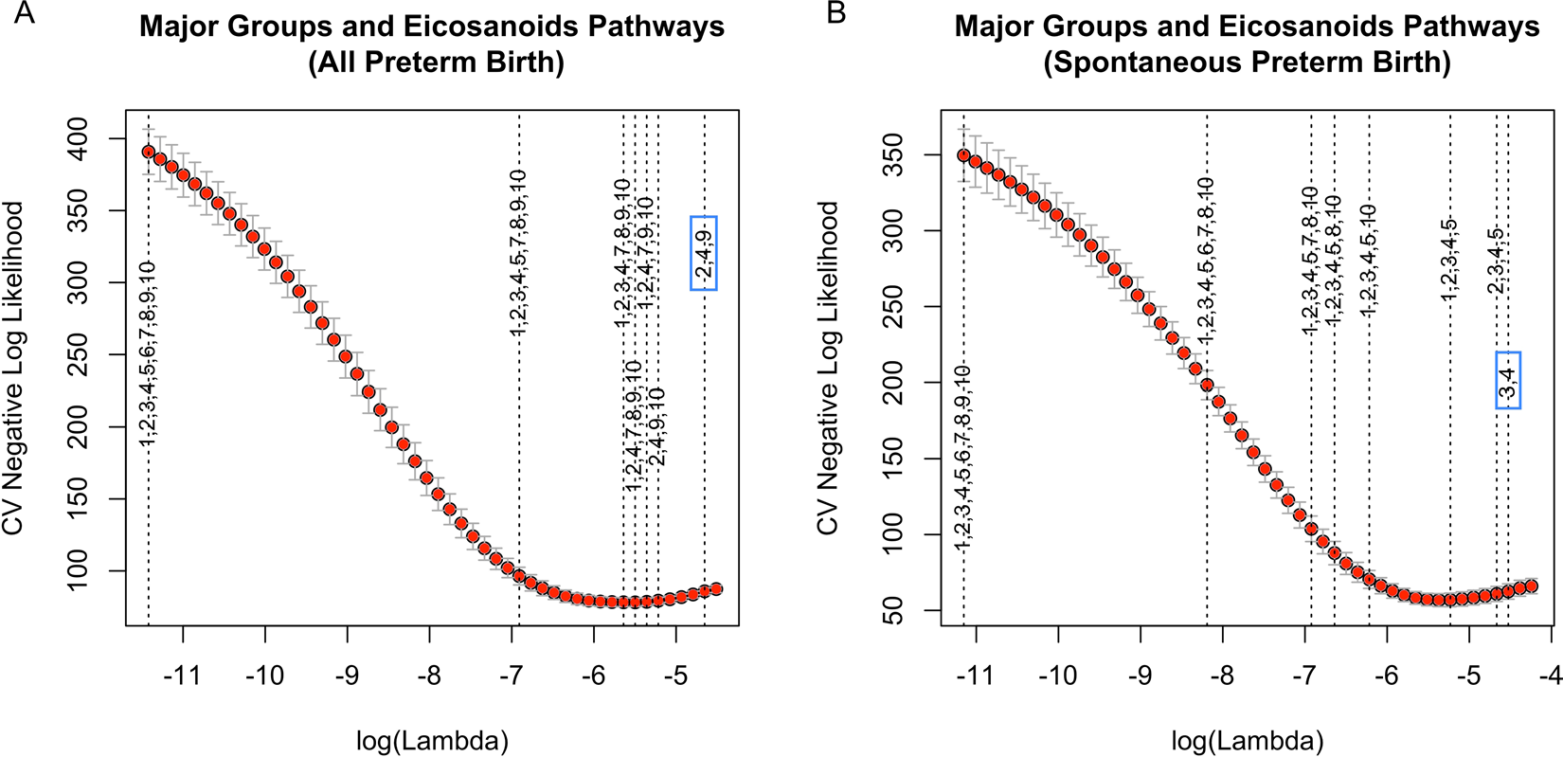
Sparse-group lasso on the combination of major groups and eicosanoids pathways for (**A**) all preterm birth and (**B**) spontaneous preterm birth. The dashed lines indicate the value of log(*λ*) corresponding to the first time the whole group of variables are excluded from the model as log(*λ*) increases. The numbers 1 to 10 on the dashed lines indicate covariates (1), cyclooxygenase pathway (2), CYP450 pathway (3), lipoxygenase pathway (4), parent compound (5), 8-isoprostane (6), DNA damage marker (7), angiogenic factors (8), protein damage markers (9), and inflammatory markers (10), respectively.

The results of sparse-group lasso on the five major groups show that when we modeled for the conditional probability of all preterm birth, angiogenic factors exited the model first (indicating least importance), and lipid damage markers and protein damage markers exited the model last (indicating greatest importance) as the penalty on the coefficients increased (Fig. 5A). For spontaneous preterm birth, lipid markers were again the last ones that exited the model (Fig. 5B). These results indicate that lipid markers contributed most to the associations of overall preterm birth and spontaneous preterm birth among the whole set of biomarkers. When the combination of major groups and eicosanoids pathways were considered (with 9 groups in total, including 4 eicosanoids pathways), biomarkers in COX pathway, LOX pathway and protein damage markers exited the model last as the tuning parameter *λ* increased for all preterm birth (Fig. 6A); CYP450 pathway and LOX pathway were the last two groups that exited the model for spontaneous preterm birth (Fig. 6B). The groups that exit the model last are most predictive of the respective preterm birth outcome.

### Prediction of preterm birth

The computed area under the Receiver Operating Characteristic (ROC) curve (AUC) is a measure of discrimination, where in our study an AUC value of 0.5 would indicate that a given combination of biomarkers has no better accuracy to predict preterm birth than chance, while an AUC value of 1 represents perfect accuracy at predicting preterm birth. While AUC values above 0.9 typically represent diagnostic tests of biomarker combinations with the greatest utility in separating cases from controls, tests with AUC values between 0.7–0.9 indicate biomarker combinations that have fair to good utility and can potentially be improved with a larger training data set or expanded measurement of additional predictive biomarkers. We reported AUC values corresponding to prediction of all preterm birth (Table 2) and spontaneous preterm birth (Table 3).

**Table 2:** Biomarkers selected by adaptive elastic-net for prediction of all preterm birth and corresponding prediction parameters estimated through adaptive elastic-net and random forest.

Biomarker	Biomarkers in the group	Biomarker(s) selected^*^	Misclassification error^†^	Sensitivity^†^	Specificity^†^	AUC
None (0)	N/A	N/A	0.40^‡^ 0.40^§^	0.57^‡^ 0.57^§^	0.61^‡^ 0.61^§^	0.66 (0.49, 0.83)^‡^ 0.62 (0.45, 0.80)^§^
All biomarkers (65)	All biomarkers listed below in this column	PGA2; 15DO12,14-PGJ2; BCPGE2; 13,14DHK-PGF2a*; RVD1; LTE4; LTB4; LA;IL-10	0.29^‖^ 0.27^§^	0.64^‖^ 1.00^§^	0.74^‖^ 0.65^§^	0.72 (0.53, 0.91)^‖^ 0.85 (0.74, 0.97)^§^
*Major group*						
DNA damage marker (1)	8-OHdG	8-OHdG	0.30^‡^ 0.26^§^	0.71^‡^ 0.64^§^	0.69^‡^ 0.78^§^	0.76 (0.61, 0.90)^‡^ 0.74 (0.56, 0.92)^§^
Angiogenic factors (2)	PGF; sFlt-1	PGF; sFlt-1	0.33^‡^ 0.41^§^	0.77^‡^ 0.69^§^	0.64^‡^ 0.56^§^	0.76 (0.60, 0.93)^‡^ 0.68 (0.50, 0.87)^§^
Protein damage markers (3)	NY; DY; CY	NY; DY; CY	0.46^‡^ 0.40^§^	0.43^‡^ 0.50^§^	0.58^‡^ 0.64^§^	0.60 (0.41, 0.79)^‡^ 0.60 (0.44, 0.77)^§^
Inflammatory markers (5)	IL-1β; IL-6; IL-10; TNF-α; CRP	IL-1β; IL-6; IL-10; TNF-α; CRP	0.28^‡^ 0.50^§^	0.57^‡^ 0.50^§^	0.78^‡^ 0.50^§^	0.73 (0.58, 0.89)^‡^ 0.57 (0.40, 0.74)^§^
Lipid damage markers (54)	8-isoprostane; all eicosanoids	15DO12,14-PGJ2; BCPGE2; RVD2; RVD1; LTE4; LTB4	0.29^‖^ 0.24^§^	0.64^‖^ 0.91^§^	0.74^‖^ 0.71^§^	0.78 (0.62, 0.94)^‖^ 0.84 (0.72, 0.96)^§^
All markers except lipid damage markers (11)	8-OHdG; PGF; sFlt-1; NY; DY; CY;IL-1β; IL-6; IL-10; TNF-α; CRP	PGF; IL-6; IL-10; TNF-α	0.31^‡^ 0.35^‖^ 0.43^§^	0.77^‡^ 0.77^‖^ 0.69^§^	0.67^‡^ 0.61^‖^ 0.53^§^	0.84 (0.71, 0.96)^‡^ 0.75 (0.56, 0.94)^‖^ 0.71 (0.54, 0.89)^§^
*Pathways of eicosanoids*						
Cyclooxygenase (15)	13,14DHK-PGD2; TXB2; PGE2; PGD2; PGA2; 15DO12,14-PGJ2; PGD3; PGE3; PGJ2; BCPGE1; BCPGE2; PGB2; 13,14DHK-PGF2a*; 9-oxoODE; PGE1	13,14DHK-PGD2; TXB2; PGE2; PGA2; 15DO12,14-PGJ2; PGJ2; BCPGE1; BCPGE2; PGB2; 13,14DHK-PGF2a*; 9-oxoODE	0.37^‡^ 0.43^‖^ 0.39^§^	0.79^‡^ 0.64^‖^ 0.71^§^	0.57^‡^ 0.64^‖^ 0.57^§^	0.73 (0.58, 0.89)^‡^ 0.58 (0.41, 0.75)^‖^ 0.65 (0.47, 0.83)^§^
CYP450 (18)	12,13-DiHOME; 9,10-DiHOME; 8,9-DHET; 8(9)-EET; 14(15)-EET; 11(12)-EET; 5(6)-EET; 12(13)-EpoME; 9(10)-EpoME; CAA; 20-HETE; 11-HETE; 16-HETE; 17-HETE; 18-HETE; 11,12-DHET; 5,6-DHET; 9S-HODE	9(10)-EpoME; 18-HETE	0.40^‡^ 0.42^‖^ 0.42^§^	0.71^‡^ 0.64^‖^ 0.43^§^	0.56^‡^ 0.56^‖^ 0.64^§^	0.63 (0.47, 0.79)^‡^ 0.68 (0.51, 0.85)^‖^ 0.56 (0.39, 0.73)^§^
Lipoxygenase (15)	RVD2; RVD1; 5-HETE; LTD4; LTE4; LTC4-ME; 13S-HODE; 5-oxoETE; 12-oxoETE; 13-oxoODE; 15-oxoETE; 15-HETE; 12-HETE; 8-HETE; LTB4	RVD1; 5-HETE; LTD4; LTE4; LTC4-ME; 13S-HODE; 13-oxoODE; 5-oxoETE; 15-oxoETE; 12-oxoETE; 12-HETE; 8-HETE; LTB4	0.33^‡^ 0.40^‖^ 0.31^§^	0.55^‡^ 0.55^‖^ 0.91^§^	0.71^‡^ 0.61^‖^ 0.61^§^	0.68 (0.49, 0.86)^‡^ 0.65 (0.45, 0.84)^‖^ 0.83 (0.69, 0.96)^§^
Parent Compound (5)	DHA; EPA; AA; αLA; LA	EPA; LA	0.38^‡^ 0.44^‖^ 0.40^§^	0.71^‡^ 0.64^‖^ 0.71^§^	0.58^‡^ 0.53^‖^ 0.56^§^	0.64 (0.47, 0.81)^‡^ 0.56 (0.37, 0.76)^‖^ 0.65 (0.48, 0.81)^§^

^*^Variables were selected by adaptive elastic-net using the training set only.

^†^The cutoff of the predicted probability used here was 0.5 (predicted probability greater than 0.5 indicates a case).

^‡^Results obtained from logistic regression models.

^§^Results obtained from random forest.

^‖^Results obtained from adaptive elastic-net.

**Table 3:** Biomarkers selected by adaptive elastic-net for prediction of spontaneous preterm birth and corresponding prediction parameters estimated through adaptive elastic net and random forest.

Biomarker	Biomarkers in the group	Biomarker(s) selected^*^	Misclassification error^†^	Sensitivity^†^	Specificity^†^	AUC
None (0)^†^	N/A	N/A	0.40^‡^ 0.33^§^	0.13^‡^ 0.50^§^	0.71^‡^ 0.71^§^	0.63 (0.40, 0.86)^‡^ 0.51 (0.24, 0.77)^§^
All biomarkers (65)^*^	All biomarkers listed below in this column	13,14DHK-PGD2; TXB2; PGD2; 15DO12,14-PGJ2; BCPGE1; 13-oxoODE; 5-oxoETE; DHA; LA; IL-10	0.37^‖^ 0.17^§^	0.75^‖^ 0.67^§^	0.60^‖^ 0.87^§^	0.69 (0.46, 0.91)^‖^ 0.79 (0.61, 0.98)^§^
*Major group*						
DNA damage marker (1)^†^	8-OHdG	8-OHdG	0.44^‡^ 0.47^§^	0.50^‡^ 0.38^§^	0.57^‡^ 0.57^§^	0.51 (0.26, 0.76)^‡^ 0.52 (0.29, 0.75)^§^
Angiogenic factors (2) ^†^	PGF; sFlt-1	PGF; sFlt-1	0.38^‡^ 0.33^§^	0.14^‡^ 0.43^§^	0.71^‡^ 0.71^§^	0.51 (0.32, 0.71)^‡^ 0.54 (0.25, 0.83)^§^
Protein damage markers (3)^†^	NY; DY; CY	NY; DY; CY	0.35^‡^ 0.30	0.38^‡^ 0.50^§^	0.71^‡^ 0.74^§^	0.53 (0.29, 0.78)^‡^ 0.64 (0.44, 0.84)^§^
Inflammatory markers (5)^†^	IL-1β; IL-6; IL-10; TNF-α; CRP	IL-1β; IL-6; IL-10; TNF-α; CRP	0.33^‡^ 0.35^§^	0.50^‡^ 0.38^§^	0.71^‡^ 0.71^§^	0.57 (0.31, 0.83)^‡^ 0.55 (0.31, 0.79)^§^
Lipid damage markers (54)^*^	8-isoprostane; all eicosanoids	PGD2; 15DO12,14-PGJ2; 5-oxoETE	0.42^‖^ 0.19^§^	0.63^‖^ 0.57^§^	0.57^‖^ 0.87^§^	0.63 (0.42, 0.84)^‖^ 0.79 (0.62, 0.96)^§^
All markers except lipid damage markers (11)^†^	8-OHdG; PGF; sFlt-1; NY; DY; CY; IL-1β; IL-6; IL-10; TNF-α; CRP	PGF; IL-6; CY; DY	0.38^‡^ 0.40^‖^ 0.36^§^	0.43^‡^ 0.43^‖^ 0.29^§^	0.66^‡^ 0.63^‖^ 0.71^§^	0.50 (0.25, 0.75)^‡^ 0.50 (0.23, 0.78)^‖^ 0.51 (0.29, 0.73)^§^
*Pathways of eicosanoids*						
Cyclooxygenase (15)^*^	13,14DHK-PGD2; TXB2; PGE2; PGD2; PGA2; 15DO12,14-PGJ2; PGD3; PGE3; PGJ2; BCPGE1; BCPGE2; PGB2; 13,14DHK-PGF2a*; 9-oxoODE; PGE1	13,14DHK-PGD2; PGE2; 15DO12,14-PGJ2; PGJ2; BCPGE1; BCPGE2; 13,14DHK-PGF2a*; PGB2	0.42^‡^ 0.28^‖^ 0.28^§^	0.50^‡^ 0.63^‖^ 0.25^§^	0.60^‡^ 0.74^‖^ 0.83^§^	0.56 (0.32, 0.79)^‡^ 0.67 (0.44, 0.90)^‖^ 0.63 (0.40, 0.87)^§^
CYP450 (18)^*^	12,13-DiHOME; 9,10-DiHOME; 8,9-DHET; 8(9)-EET; 14(15)-EET; 11(12)-EET; 5(6)-EET; 12(13)-EpoME; 9(10)-EpoME; CAA; 20-HETE; 11-HETE; 16-HETE; 17-HETE; 18-HETE; 11,12-DHET; 5,6-DHET; 9S-HODE	5(6)-EET; 12(13)-EpoME; 11,12-DHET	0.33^‡^ 0.26^‖^ 0.23^§^	0.38^‡^ 0.63^‖^ 0.50^§^	0.74^‡^ 0.77^‖^ 0.83^§^	0.66 (0.46, 0.86)^‡^ 0.74 (0.52, 0.96)^‖^ 0.73 (0.51, 0.95)^§^
Lipoxygenase (15)^*^	RVD2; RVD1; 5-HETE; LTD4; LTE4; LTC4-ME; 13S-HODE; 5-oxoETE; 12-oxoETE; 13-oxoODE; 15-oxoETE; 15-HETE; 12-HETE; 8-HETE; LTB4	5-HETE; LTD4; LTC4-ME; 13-oxoODE; 5-oxoETE; 8-HETE; LTB4	0.51^‡^ 0.44^‖^ 0.27^§^	0.71^‡^ 0.63^‖^ 0.43^§^	0.43^‡^ 0.51^‖^ 0.80^§^	0.62 (0.38, 0.85)^‡^ 0.60 (0.35, 0.85)^‖^ 0.82 (0.68, 0.96)^§^
Parent Compound (5)^†^	DHA; EPA; AA; αLA; LA	EPA; AA; αLA; LA	0.35^‡^ 0.26^‖^ 0.23^§^	0.25^‡^ 0.50^‖^ 0.50^§^	0.74^‡^ 0.80^‖^ 0.83^§^	0.59 (0.39, 0.78)^‡^ 0.67 (0.43, 0.91)^‖^ 0.63 (0.39, 0.86)^§^

^*^Variables were selected by adaptive elastic-net using the training set only.

^†^The cutoff of the predicted probability used here was 0.5 (predicted probability greater than 0.5 indicates a case).

^‡^Results obtained from logistic regression models.

^§^Results obtained from random forest.

^‖^Results obtained from adaptive elastic-net.

**Figure 7:**
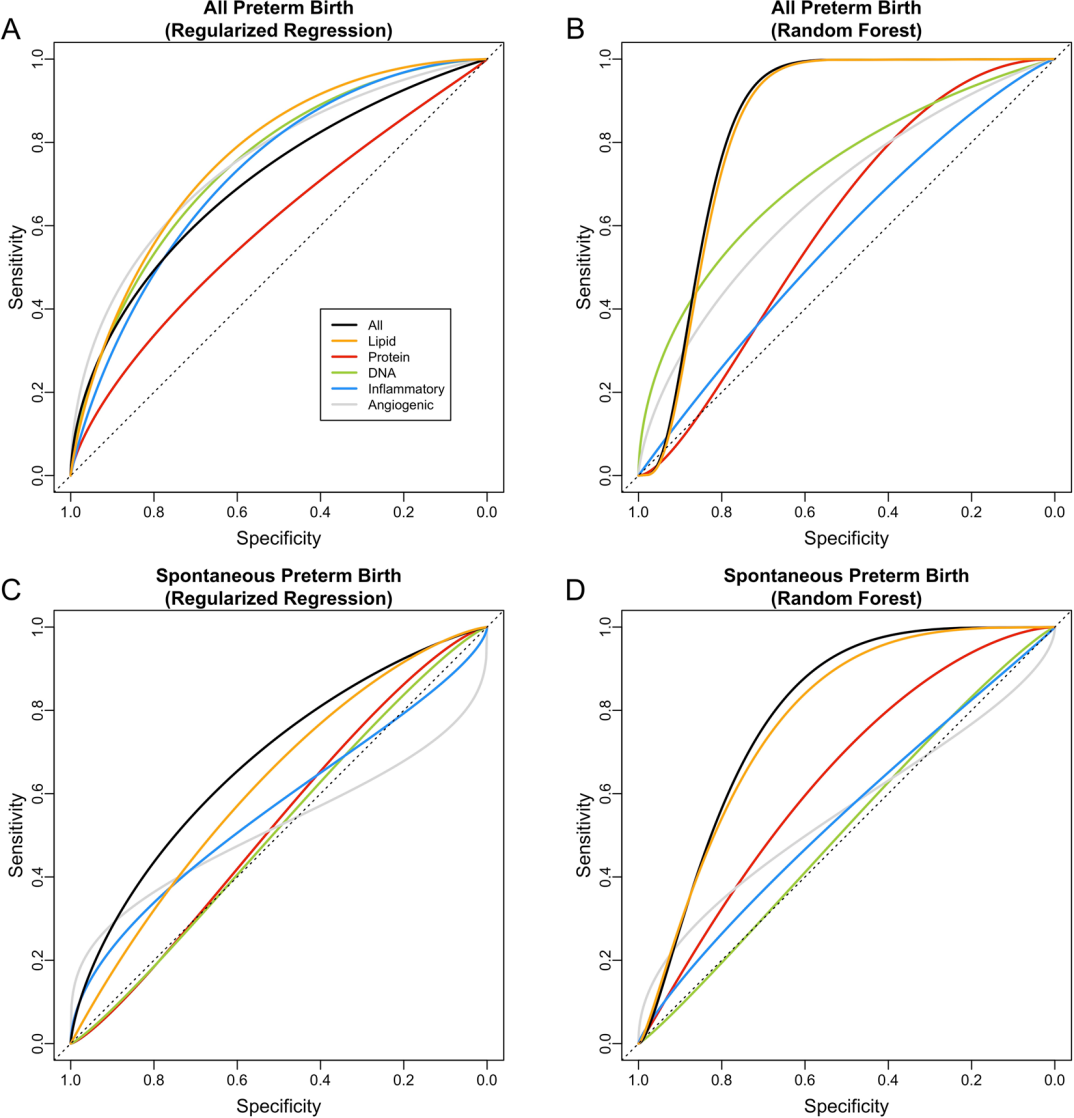
The Receiver Operating Characteristic (ROC) curve for the selected model for major groups. The area under the ROC curve for each group is: (**A**) logistic regression or adaptive elastic-net for all preterm: all (0.72 [0.53, 0.91]), lipid (0.78 [0.62, 0.94]), angiogenic factors (0.76 [0.60, 0.93]), protein (0.60 [0.41, 0.79]), DNA (0.76 [0.61, 0.90]), inflammatory (0.73 [0.58, 0.89]); (**B**) random forest for all preterm: all (0.85 [0.74, 0.97]), lipid (0.84 [0.72, 0.96]), angiogenic factors (0.68 [0.50, 0.87]), protein (0.60 [0.44, 0.77]), DNA (0.74 [0.56, 0.92]), inflammatory (0.57 [0.40, 0.74]); (**C**) logistic regression or adaptive elastic-net for spontaneous preterm: all (0.69 [0.46, 0.91]), lipid (0.63 [0.42, 0.84]), angiogenic factors (0.51 [0.32, 0.71]), protein (0.53 [0.29, 0.78]), DNA (0.51 [0.26, 0.76]), inflammatory (0.57 [0.31, 0.83]); (**D**) random forest for spontaneous preterm: all (0.79 [0.61, 0.98]), lipid (0.79 [0.62, 0.96]), angiogenic factors (0.54 [0.25, 0.83]), protein (0.64 [0.44, 0.84]), DNA (0.52 [0.29, 0.75]), inflammatory (0.55 [0.31, 0.79]).

**Figure 8:**
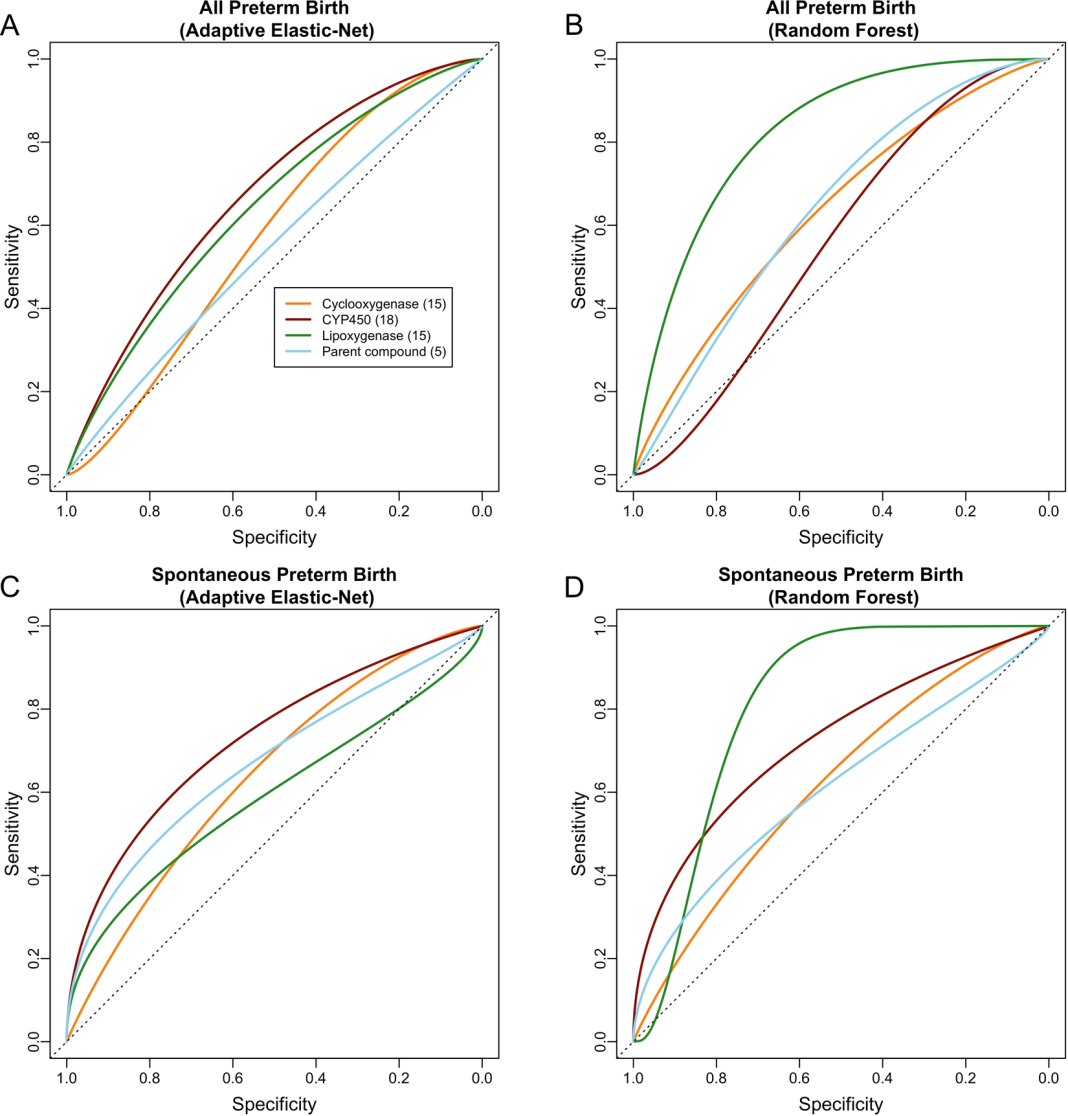
The Receiver Operating Characteristic (ROC) curve for the selected model for biological pathways of eicosanoids. The area under the ROC curve for each group is: (**A**) adaptive elastic-net for all preterm: cyclooxygenase (0.58 [0.41, 0.75]), CYP450 (0.68 [0.51, 0.85]), lipoxygenase (0.65 [0.45, 0.84]), parent compound (0.56 [0.37, 0.76]); (**B**) random forest for all preterm: cyclooxygenase (0.65 [0.47, 0.83]), CYP450 (0.56 [0.39, 0.73]), lipoxygenase (0.83 [0.69, 0.96]), parent compound (0.65 [0.48, 0.81]); (**C**) adaptive elastic-net for spontaneous preterm: cyclooxygenase (0.67 [0.44, 0.90]), CYP450 (0.74 [0.52, 0.96]), lipoxygenase (0.60 [0.35, 0.85]), parent compound (0.67 [0.43, 0.91]); (**D**) random forest for spontaneous preterm: cyclooxygenase (0.63 [0.40, 0.87]), CYP450 (0.73 [0.51, 0.95]), lipoxygenase (0.82 [0.68, 0.96]), parent compound (0.63 [0.39, 0.86]).

According to Table 2 and Fig. 7A,B, when all preterm birth was considered, among the five major groups of markers, lipid markers provided better separation of cases and controls (adaptive elastic-net AUC = 0.78 [0.62, 0.94], random forest AUC = 0.84 [0.72, 0.96]) than the other four groups (which collectively had AUC values ranging from 0.57–0.76), and such performance was comparable to using all biomarkers (adaptive elastic-net AUC = 0.72 [0.53, 0.91], random forest AUC = 0.85 [0.74, 0.97]). The misclassification errors yielded by lipid markers for adaptive elastic-net (0.29) and random forest (0.24) were among the smallest across the five major groups. The results for the other four major groups were not consistent across different training methods, and such discrepancy indicated either a nonlinear and complex relationship between the corresponding biomarkers and outcome variables or potentially random chance (Fig. 7A,B). Among the three eicosanoid enzymatic pathways, the LOX pathway gave the best overall performance in terms of misclassification error and AUC (Table 2 and Fig. 8A,B). In particular, LOX pathway had higher AUC (0.83 [0.69, 0.96]) and sensitivity (0.91) than the other three groups when we used random forest to train the prediction model (Table 2 and Fig. 8A,B).

When spontaneous preterm birth was considered, the group of lipid markers again gave the largest AUC (adaptive elastic-net AUC = 0.63 [0.42, 0.84], random forest AUC = 0.79 [0.62, 0.96]) and sensitivity (adaptive elastic-net sensitivity = 0.63, random forest sensitivity = 0.57) among the five major groups for each training method (Table 3 and Fig. 7C,D). The adaptive elastic-net AUC of the groups of DNA damage markers, angiogenic factors, and inflammatory markers ranged from 0.51 to 0.57, which indicated that the performance of these three groups was barely (if any) better than random guess (Table 3 and Fig. 7C,D). According to Table 3 and Fig. 8C, biomarkers involved in the CYP450 pathway generally performed better than those involved in the other three pathways in classifying spontaneous preterm birth when we used adaptive elastic-net to train the model. However, the LOX pathway outperformed the CYP450 pathway when we used random forest (Table 3 and Fig. 8D), which indicates that potential nonlinear relationships may exist between spontaneous preterm birth and biomarkers in the LOX pathway that were not captured by adaptive elastic-net.

## Discussion

In this study, we made two primary contributions: (1) integrated cutting edge machine learning methods and multiple inferential tools (hypothesis testing, variable selection, and prediction) to analyze an extensive panel of biomarkers related to inflammation, oxidative stress, and angiogenesis, in association with preterm birth and its subtypes; and (2) contextualized associations with preterm birth by grouped analyses of biomarker categories and enzymatic pathway groups for eicosanoids. To our knowledge, this is the first study to investigate eicosanoids and associated biomarkers to this degree of metabolite coverage during pregnancy in association with overall preterm birth and its subtypes. Several biomarkers were associated with overall preterm birth in single biomarker models, and adaptive elastic-net identified five biomarkers (PGF, LA, LTB4, 5(6)-EET, and 15DO12,14-PGJ2) to be most predictive of overall preterm birth. The overall preterm birth outcome contains multiple constituent sub-pathologies, therefore interpretation of these associations is more crude compared to focusing on preterm birth subtypes. Once we disaggregated cases by preterm birth subtypes, we learned that many of the effect estimates were largely driven by spontaneous preterm birth, where adaptive elastic-net identified four biomarkers (5-oxoETE, RVD1, 5(6)-EET, and 15DO12,14-PGJ2) to be most predictive. Whereas for preterm birth associated with aberrant placentation, adaptive elastic-net identified two biomarkers with protective associations (PGF and LTB4). Prediction of preterm birth was most accurate with the lipid biomarkers using both adaptive elastic-net and random forest. When focusing solely on the lipid biomarkers, adaptive elastic-net showed greater accuracy in preterm birth prediction among metabolites of the CYP450 pathway, while random forest reported metabolites of the LOX pathway to be more accurate. Overall, these findings provide insight on prioritization of biomarker groups for the prediction of preterm birth, and future studies should explore CYP450 and LOX metabolites to validate these findings.

The biomarkers that adaptive elastic-net identified as most predictive for spontaneous preterm birth are all eicosanoid metabolites, with each of the three enzymatic pathways and parent compounds being represented. 5-oxoETE is a polyunsaturated fatty acid product derived from AA processing via the LOX pathway. Receptors for 5-oxoETE are highly expressed in peripheral leukocytes, lungs, kidney, liver, spleen, and placenta^16,17^. The impact that 5-oxoETE has on target sites can vary by cell type, but broadly involve intracellular changes that lead to heightened chemotaxis – an integral process that attracts immune cells to target tissues to drive the inflammatory process^18^. 5(6)-EET is a fatty acid metabolite of AA produced through the CYP450 pathway and commonly originates from the liver, kidneys, and vascular endothelium^19^. Importantly, 5(6)-EET is a modulator of cardiovascular function and predominantly acts as an anti-inflammatory vasodilator^19,20^. We observed protective associations between 5(6)-EET and spontaneous preterm birth, which aligns with the predominant paradigm of an anti-inflammatory and anti-hypertensive profile. However, other studies have observed higher plasma and placental concentrations of 5(6)-EET or total EET in women with preeclampsia compared to normotensive women^19,21^ and non-pregnant women^22^. Another study found lower concentrations of 5(6)-EET in amnion of women with preeclampsia compared to laboring women that had a cesarean section^23^. Differences between studies underline potential nuances to 5(6)-EET activity, potentially masked by our uncertainty of concentrations at target tissues at the maternal-fetal interface.

The prostaglandin 15DO12,14-PGJ2 has been shown to elicit anti-inflammatory properties through inhibition of the nuclear transcription factor NFκ-B^24^. Although we observed positive associations between 15DO12,14-PGJ2 and spontaneous preterm birth, several pro-inflammatory prostaglandins (PGB2, 13,14DHK-PGF2a, PGE2, and 13,14DHK-PGD2) were also positively associated with spontaneous preterm birth. 15DO12,14-PGJ2 may likely be up-regulated during inflammation to help with the resolution of inflammation, thereby partly explaining why we observed positive associations with spontaneous preterm birth. A similar conclusion may be drawn from the positive association we observed between spontaneous preterm birth and the anti-inflammatory molecule RVD1, which belongs to the group of compounds called resolvins and is a LOX pathway product of DHA^14,25^. Interestingly, our findings suggest that both pro- and anti-inflammatory fatty acid metabolites are associated with increased risk for overall and spontaneous preterm birth, emphasizing the utility of evaluating these biomarkers collectively as a combination of predictors to enhance the classification of preterm birth cases early in pregnancy.

Among the predictors that were linked to preterm birth associated with aberrant placentation, adaptive elastic net identified PGF and LTB4 as most predictive, both of which are protective for this type of preterm birth. PGF is a growth factor that promotes angiogenesis in part by stimulating cellular migration and recruitment^26^. Previous studies observed lower levels of PGF among women with preeclampsia – one requisite for preterm birth associated with aberrant placentation – indicating that higher concentrations of PGF may potentially be protective of this type of preterm birth^26^. LTB4 is a leukotriene metabolized from AA through the LOX pathway, and has pro-inflammatory properties through stimulating vasoconstriction, platelet aggregation, and cellular migration of immune cells^27^. Although LTB4 has been previously reported to increase the risk for preeclampsia^27^, we observed a protective relationship between LTB4 and preterm birth associated with aberrant placentation. One possible explanation is that LTB4 elevation may potentially be a proxy for increased anti-inflammatory biomarkers, given evidence that LTB4 concentrations can remain high before and after onset of preeclampsia^27^. Conversely, this observation, alongside numerous null associations, may be due to underpowered sample size of this type of preterm birth in our study.

Very few studies have investigated circulating maternal eicosanoids and lipid biomarkers in association with preterm birth. One case control study of preterm birth (n_cases_ = 37, n_controls_ = 37) observed higher concentrations of AA in plasma collected at delivery from women who delivered preterm compared to AA in plasma collected at 34 weeks gestation from women that delivered term, which is consistent with the increased risk we observed between AA and spontaneous preterm birth^28^. Another study evaluated differences in PGF2α and PGE2 by preterm status (n_cases_ = 37, n_controls_ = 34) and found lower levels of both in placenta and amnion samples from cases collected at delivery, but did not find significant differences in maternal plasma^29^. A separate case-control study (n_cases = _50, n_controls_ = 50) found lower levels of PGF2α measured in serum collected at the onset of active labor in preterm birth cases compared to controls^30^. However, another case-control study of preterm birth (n_cases_ = 57, n_controls_ = 624) did not observe notable differences in urinary concentrations of PGF2α^31^. Although we did not measure PGF2α, we did find higher concentrations of its metabolite 13,14DHK-PFG2α among spontaneous preterm birth cases. Ultimately there are few existing studies for comparisons, however our findings are largely consistent with current frameworks for biological mechanisms of eicosanoids.

The findings of this study can inform future screening tests aimed at detecting high-risk groups for preterm birth and its subtypes. Our study provides evidence that utilizing multiple biomarkers simultaneously to predict preterm birth is more accurate than using single biomarkers. However there is evidence of differentiation in predictive capacity across categories of biomarkers. In our study sample, biomarkers of lipid metabolism have good accuracy in identifying cases of overall and spontaneous preterm birth. Specifically, the eicosanoid panel used in this study had comparable predictive capacity compared to using the full combination of inflammatory, oxidative stress, and eicosanoid biomarkers. Adaptive elastic-net also selected lipid biomarkers in higher frequency than all other categories of biomarkers. These findings underline the predictive utility of lipid biomarkers and this should be replicated for consistency in an independent sample. Focusing in on eicosanoids from specific pathways can be useful when resources are limited, especially given that measuring extensive panels of signaling molecules can be cost-prohibitive. Future studies in independent samples could potentially utilize estimations from our present study to replicate prediction of preterm birth using any combination of eicosanoids that were presented in this study.

Among the eicosanoids, metabolites from the LOX and CYP450 pathways had greater performance of overall and spontaneous preterm birth classification. Animal models indicate that the LOX pathway eicosanoid products are collectively involved inflammation, vascular remodeling, and renal function^12,32^. For example, mouse models with knockouts of LOX enzymes resulted in impaired implantation, indicating an important role for LOX products early in pregnancy^33^. Similarly, eicosanoids produced through CYP450 pathway also contribute to vascular remodeling, with a hypothesized link to hypertension and inflammation^34^. For instance, in rat models, disruption in the production of eicosanoids from the CYP450 pathway has been shown to alter myometrium contractile activity^35^ and lead to the development of hypertension and renal dysfunction^36^. Thus, LOX and CYP450 eicosanoid products as whole groups can be useful in predicting broad pathological precursors to preterm birth; whereas analysis of disruption in any single metabolite may only provide limited insight due to potential compensatory roles of multiple metabolites produced from the same pathway.

This study contains several limitations that should be discussed to improve future studies. First, our study had low numbers of cases, especially as we disaggregated to preterm birth subtypes. We may have been underpowered to observe smaller effect estimates with some of the biomarkers that we measured. The greater count of spontaneous preterm birth cases compared to preterm birth associated with aberrant placentation may have biased the results of overall preterm birth towards the biological mechanisms underlying spontaneous preterm birth. Additionally, samples were collected between 2006 and 2008, therefore biomarkers in our samples may be susceptible to degradation over long periods of storage. However, rigorous storage protocols for freezing and preserving samples partially ameliorate this problem. Furthermore, the measurement error bias associated with long storage periods would likely lead to attenuation of research findings towards null, rather than create false positives. Another limitation in our study was the cross-sectional study design and we were limited to a single time point measurement of biomarkers, therefore we were unable to evaluate time-course concentrations of individual biomarkers during pregnancy. One previous repeated measures study of select eicosanoids in adult women (n = 9) observed low intra-individual variability across eight repeated blood measurements^37^. Conversely, another study of adult men (n = 19) observed high intra-individual variability in two repeated measurements of blood eicosanoids^38^. In the LIFECODES cohort, we have also leveraged repeated measurements of the inflammatory biomarkers (CRP and cytokines), and observed moderate intra-individual variability^9^. These results provide preliminary evidence that although single time point measurements may have good utility in some study samples, repeated measurements would be advantageous when intra-individual variability is moderate or high. Future studies should consider measuring these biomarkers at multiple study visits that range across each trimester of pregnancy and conduct longitudinal analyses to determine if specific periods of gestation have greater predictive capacity.

Since we conducted multiple comparisons, some of the single biomarker models may be at risk for false discoveries. However, the biomarkers that we discussed in depth that were selected by adaptive elastic-net circumvent the drawbacks of multiple comparisons since it evaluates all biomarkers in a single predictor matrix simultaneously rather than conducting multiple tests. We are also limited by the fact that we measured for circulating plasma or urinary biomarkers, which provides limited insight on the physiology occurring locally at the maternal-fetal interface and other target tissues. It is critical that future studies consider measuring endogenous biomarkers in multiple biological media across various time points in pregnancy. Among the biomarkers that we measured, our study focused predominantly on biomarkers measured in plasma samples. Other less invasive media such as urine, fingernail clippings, hair samples, and oral swabs can also be explored for potential predictive biomarkers. Follow up studies need to explore these additional biological media and test for their predictive capacity in observational studies. Finally, our study measured several biomarkers from the repertoire of lipid biomarkers; however we were limited in our measurement of biomarkers from the other major groups (immune, protein oxidation, growth factors). The lack of representation of biomarkers from these other major groups may also be contributing to some degree to our observations of higher prediction capacity among lipid biomarkers compared to immune, protein oxidation, and growth factor biomarkers.

Despite these limitations, our study contains several strengths to highlight. First, the study was conducted in a well-characterized birth cohort. Further, this study used high-sensitivity instrumentation to measure an extensive panel of biomarkers to assess predictors of preterm birth. Our findings provide insight on the utility of several biomarkers and groups of biomarkers to predict preterm birth. We integrated multiple statistical methods for high-dimensional data analysis that can be applied in future studies seeking to analyze a large amount of biomarkers. Another strength is that our differentiation of preterm birth subtypes provides greater clinical and physiological insight on the etiology of preterm birth. Overall our study presents new applications of statistical frameworks within biological context for future studies that seek to utilize maternal biomarkers to predict preterm birth.

In conclusion, this study demonstrated that lipid biomarkers are good predictors of overall and spontaneous preterm birth. Our findings attempt to contribute a first step at prioritization of biomarker categories and eicosanoid enzymatic pathways for future studies that seek to predict preterm birth and inform precision health studies. Among eicosanoids, LOX and CYP450 products have the greatest performance of identifying overall and spontaneous preterm birth. We recommend replication studies to evaluate the consistency of our findings, and conclude that lipid biomarkers may have good utility in clinical settings to predict preterm birth.

## Methods

### Study population

The LIFECODES prospective birth cohort enrolled approximately 1,600 pregnant women between 2006 and 2008 at the Brigham and Women’s Hospital in Boston, MA. Participants were between 20 and 46 years of age, recruited at less than 15 weeks gestation at the initial study visit, and attended up to 4 study visits (targeted at median 10, 18, 26, and 35 weeks gestation) at which point they provided urine and blood specimens. At the initial study visit, questionnaires were administered to collect demographic and health-related information. Among participants recruited in the LIFECODES cohort, 1,181 participants were followed to term, and delivered live, single infants. This present study was cross-sectional and focused on urine and plasma samples collected at one study visit occurring between 23.1 and 28.9 weeks gestation (median = 26 weeks). The study sample included 58 women who delivered preterm (<37 weeks gestation), and 115 randomly selected women who delivered after 37 weeks gestation. Among the cases, there were 31 spontaneous preterm births and 25 preterm births associated with aberrant placentation, and 2 cases that could not be sufficiently categorized as either. This study received institutional review board (IRB) approval from the Brigham and Women’s Hospital and all participants provided written informed consent. All of the methods within this study were performed in accordance with the relevant guidelines and regulations approved by the IRB. Additional details regarding recruitment and study design can be found elsewhere^39,40^.

### Biomarker analysis

Spot urine samples were collected and stored at −80 °C. Approximately 10 mL of blood was collected using ethylenediaminetetraacetic acid plasma tubes and temporarily stored at +4 °C for less than 4 hours. Afterwards, blood was centrifuged for 20 minutes and stored at −80 °C. Urine and plasma were subsequently analyzed for endogenous biomarkers. Supplemental Fig. 1 illustrates each individual biomarker that we analyzed and the media (urine vs. plasma) in which they were measured. We measured for the immunological biomarkers CRP, interleukin-1β [IL-1β], interleukin-6 [IL-6], tumor necrosis factor-α [TNF-α], and IL-10 in plasma. These biomarkers were quantified at the Cancer Center Immunology Core at the University of Michigan (Ann Arbor, MI, USA). CRP was measured using a DuoSet enzyme-linked immunosorbent assay (ELISA) (R&D Systems, Minneapolis, MN). The remaining cytokines were simultaneously measured using the Milliplex MAP High Sensitivity Human Cytokine Magnetic Bead Panel (EMD Millipore Corp., St. Charles, MO). Further details on detection rates and assay sensitivity were previously described^9^. We also measured angiogenic factors PGF and soluble fms-like tyrosine kinase-1 [sFlt-1] in plasma using the ARCHITECT immunoassays by Abbott Laboratories (Abbott Park, IL).

The expanded panel of lipid biomarkers included 53 eicosanoids and lipid metabolites measured using a 6490 Triple Quadrupole mass spectrometer (Agilent, New Castle, DE, USA), set to a targeted multiple reaction monitoring mode. Eicosanoids were identified based on metabolite-specific fragmentation and corresponding retention time. Further details on instrument parameters and quality control can be found elsewhere^41^. We measured for five oxidative stress biomarkers. The biomarkers total 8-isoprostane [8-IP] and 8-hydroxydeoxyguanosine [8-OHdG] were measured in urine samples by Cayman Chemical (Ann Arbor, MI). Urine samples were hydrolyzed for deconjugation of 8-IP and then purified through affinity column chromatography prior to measurement with enzyme immunoassay. 8-OHdG was measured with direct dilution into buffer without purification using an enzyme immunoassay.

Three protein oxidative stress markers were measured in plasma samples, including 3-nitrotyrosine [NY], 3-chlorotyrosine [CY], and *o*,*o’*-dityrosine [DY]. To quantify these markers, plasma protein was first precipitated from plasma samples and diluted with a phosphate buffer. Plasma samples were then delipidated, spiked with isotopically labeled internal standards, and hydrolyzed for 24 hours. The processed plasma samples were then analyzed for each protein oxidation marker via liquid chromatography electrospray ionization tandem mass spectrometry^42^.

### Outcome variables

We defined overall preterm birth as delivery prior to 37 weeks gestation. Among the preterm birth cases, we categorized a subset as spontaneous preterm births based on the following clinical criteria: (1) preterm premature rupture of the membranes (PPROM), or (2) spontaneous preterm labor^5^. Clinically, we defined another subset of preterm birth cases as those associated with aberrant placentation using the following criteria: (1) preterm birth resulting from the presentation of preeclampsia (encompassing onset of hypertension and proteinuria), or (2) intrauterine growth restriction (IUGR; <10^th^ percentile in fetal weight for gestational age) during pregnancy^5^.

### Association of preterm birth with single biomarkers

In this study, we evaluated the associations of overall preterm birth, in addition to spontaneous preterm birth, and preterm birth associated with aberrant placentation subtypes with 65 endogenous biomarkers of interest. The subsequent analyses were restricted to individuals who had all information on covariates and biomarkers available: *n*_cases+controls_ = 138 for all preterm, *n*_cases+controls_ = 121 for spontaneous preterm and *n*_cases+controls_ = 110 for preterm birth associated with aberrant placentation. We first investigated the effects of single endogenous biomarkers on overall, spontaneous, and preterm birth associated with aberrant placentation. Measurements were log-transformed and then standardized with mean of 0 and standard deviation of 1. The effects were estimated from the model:

$$\begin{array}{c}{\rm{logit}}\,P({\boldsymbol{Y}}|{\boldsymbol{M}},{\boldsymbol{Z}})={\theta }_{0}+{\theta }_{m}{\boldsymbol{M}}+{\boldsymbol{Z}}{{\boldsymbol{\theta }}}_{{\boldsymbol{z}}}\,(Model\,1)\end{array}$$

where ***Y*** denotes an *n* × 1 vector of the outcome variable in separate logistic regression models where each preterm birth outcome was individually modeled to test for associations compared to term births. ***M*** is an *n* × 1 vector that represents the biomarker of interest. The covariate matrix ***Z*** contained age at the initial visit, race, education level, health insurance provider, and maternal BMI at the initial study visit. Age is a continuous variable and the rest of the covariates were categorical. ***Z*** further included specific gravity if ***M*** is a urinary biomarker (8-OHdG and 8-IP). To account for multiple testing and control the FDR, we applied the Benjamini-Hochberg procedure to the set of p-values obtained from *Model 1* with FDR being 0.1 for each definition of outcome^43^. We also performed a sensitivity analysis by examining associations in unadjusted models.

### Association of preterm birth with individual and grouped biomarkers

One of our study goals was to determine which of the 65 biomarkers were contributing most to associations with preterm birth in a multiple biomarker model:

$${\rm{logit}}\,P({\boldsymbol{Y}}|{\boldsymbol{M}},{\boldsymbol{Z}})={\beta }_{0}+{\boldsymbol{M}}{{\boldsymbol{\beta }}}_{M}+{\boldsymbol{Z}}{{\boldsymbol{\beta }}}_{z}\,(Model\,2)$$

where ***Y*** and ***Z*** are defined in the same way as for *Model 1*, while ***M*** is now an *n* × *m* matrix of the biomarkers of interest with *m* being the number of biomarkers considered in *Model 2*. We applied adaptive elastic-net^44,45^, a regularized regression approach, to each of the three outcome definitions separately using the R package *gcdnet*. The estimates of the *p* × 1 vector ***β*** = (*β*_0_, ***β***_*M*_, ***β***_*z*_)^*T*^ were obtained by solving:

$${\rm{\arg }}\,\mathop{\min }\limits_{{\boldsymbol{\beta }}}\{\frac{1}{n}h(y,m,z;{\boldsymbol{\beta }})+\frac{{\lambda }_{2}}{2}{{\boldsymbol{\beta }}}_{2}^{2}+{\lambda }_{1}\mathop{\sum }\limits_{j=1}^{p}{\hat{w}}_{j}|{\beta }_{j}|\}$$

which uses both lasso^46^ and ridge^47^ type penalty to perform variable selection with a correlated set of candidate predictors. The function *h*(*y*,*m*,*z*;***β***) is an assumed loss function, and the the adaptive weights ${\hat{w}}_{j}$’s were constructed using the coefficient estimates obtained from regular elastic-net. We let the parameter for the ${\ell }_{2}$ penalty (*λ*_2_) take values on a grid from 0 to 2, in increments of 0.1. Furthermore, we forced the covariates *Z* to always be included in the model. For each fixed *λ*_2_, the function automatically computes the solutions for a fine grid of *λ*_1_’s, the ${\ell }_{1}$ regularization parameter, assuming a Huberized squared hinge loss. We selected the combination of *λ*_1_ and *λ*_2_ that yielded the smallest 10-fold cross-validated misclassification error and used their corresponding coefficient estimates as adaptive weights.

In addition to analyzing each of the 65 biomarkers individually, we further sought to evaluate associations within biological context. To accomplish this, we organized the biomarkers into five major groups (Table 2): (1) DNA damage marker (8-OHdG), (2) angiogenic factors (PGF, sFlt-1), (3) protein damage markers (NY, CY, and DY), (4) immune biomarkers (CRP, IL-1β, IL-6, TNF-α, IL-10), and (5) lipid biomarkers (8-IP and all eicosanoids). We also sought to evaluate the 53 eicosanoids by enzymatic pathways, therefore we further organized the eicosanoids according to the three enzymatic pathways derived from previous studies (LOX, CYP450, or COX)^41^, or if they are parent compounds (Table 2). To investigate how individual groups were associated with preterm birth, we applied sparse-group lasso to the five major groups of biomarkers that we created, which performs selection among these whole groups to determine which groups are most predictive of preterm birth^48^. The coefficient estimates are solutions to:

$$\arg \,\mathop{min}\limits_{{\boldsymbol{\beta }}}\{\,-\,\frac{1}{n}\,\log \,L({\boldsymbol{\beta }})+(1-\alpha )\lambda \mathop{\sum }\limits_{k=1}^{q}\sqrt{{p}_{k}}\parallel {{\boldsymbol{\beta }}}^{(k)}{\parallel }_{2}+\alpha \lambda \parallel {\boldsymbol{\beta }}{\parallel }_{1}\}$$

where *L*(***β***) is the likelihood function for *Model 2*, *q* is the total number of groups considered (in this case *q* = 5), *p*_*k*_ is the number of predictors in group *k*, and ∥_1_ and ∥_2_ are L1 and L2 norm, respectively. The mixing parameter *α* and tuning parameter *λ* together controls both “groupwise sparsity” and “within group sparsity” of non-null biomarkers. We set *α* to be 0.95, which assumes strong overall sparsity but encourages grouping^48^. In a secondary sparse-group lasso implementation, we split the major group of lipid damage markers into five subgroups, which included groupings for the three enzymatic pathways, lipid parent compounds, and 8-IP on its own (*q* = 9). Since current R implementation of sparse-group lasso does not support the specification of non-penalized coefficients, we treated the group of covariates as an additional group^48^. The relative importance of the associations was evaluated based on the sequence of the path in which each group exits the model as the tuning parameter *λ* increased (the group exiting last being the most important).

### Prediction of preterm birth

We evaluated the performance of all and grouped endogenous biomarkers in predicting preterm birth. Subjects were randomly divided into training set and validation set with a ratio of 70:30 for the respective comparison of overall, spontaneous, and preterm birth associated with aberrant placentation vs. term deliveries. The training set was resampled using the Synthetic Minority Over-sampling Technique^49^ to achieve balance between cases and controls, which may improve the prediction of minority class (in this case preterm birth). We used three different prediction algorithms using: (1) logistic regression, (2) adaptive elastic-net, and (3) random forest. First, we fitted a standard logistic regression model (*Model 2*) with the coefficients being estimated by maximum likelihood to the resampled training data for all biomarkers organized by each major group, and each eicosanoid enzymatic pathway. This method serves as a benchmark for evaluating the performance in prediction. The group of lipid damage biomarkers (all eicosanoids and 8-IP) was excluded for this approach because the maximum likelihood estimates obtained from logistic regression were unstable due to the high dimension of predictors. Second, we trained the model by applying adaptive elastic-net to the training set for each major group of biomarkers, the process of which was the same as what was done on the whole data set. Building on this framework, we also applied the same adaptive elastic-net method to the training set where we focused solely on the lipid biomarkers differentiating the three enzymatic pathways and the parent compounds. Finally, going beyond parametric regression methods, we trained the model using random forest for all groups considered, where the number of variables randomly sampled at each split for the random forest method was determined using 10-fold cross validation. The logistic regression prediction approach is limited and becomes unstable in the presence of multicollinearity and high-dimensionality of covariates and predictors. The adaptive elastic-net prediction method has improved performance in the presence of multi-collinearity, however it is unable to account for non-linear relationships between predictors and outcome variables. Finally, the random forest prediction method is able to account for non-linear relationships and interactions among predictors.

For all three prediction approaches, we obtained the predicted probability of the outcome of interest for each subject in the validation set using the optimal parameters determined by the training set. Subjects were classified as cases if their predicted probabilities were greater than 0.5 and controls otherwise. We calculated the misclassification error, sensitivity and specificity, the latter two of which are defined as the probability of correctly identifying the participants with and without preterm delivery, respectively. Over fitting can be a possible concern in the logistic regression prediction method; however over fitting is not a major concern in both adaptive elastic-net and random forest prediction methods. We also examined the ROC curve and computed the AUC to assess the performance of the final model.

### Ethical approval and informed consent

This study received institutional review board approval from the Brigham and Women’s Hospital and all participants provided written informed consent.

## Supplementary information

Supplementary material

## Data Availability

Due to the sensitive nature of biological samples and demographic variables of the human subjects dataset, the data used for this manuscript should remain confidential and not publicly shared to protect human subjects.
