# Supplementary material for: Prediction and associations of preterm birth and its subtypes with eicosanoid enzymatic pathways and inflammatory markers
Source: Sci Rep. 2019 Nov 19;9:17049. doi: 10.1038/s41598-019-53448-z (PMC6863859; doi:10.1038/s41598-019-53448-z)
Supplement: Supplementary file 1 — Supplementary material [file 41598_2019_53448_MOESM1_ESM.pdf]

**Supplementary Material**

**Title:** Prediction and associations of preterm birth and its subtypes with eicosanoid enzymatic pathways and inflammatory markers

**Authors:** Max T. Aung[1], Youfei Yu[2], Kelly K. Ferguson[3], David E. Cantonwine[4], Lixia Zeng[5], Thomas F. McElrath[4], Subramaniam Pennathur[5,6,7], Bhramar Mukherjee[2,8], John D. Meeker[1]

[1]Department of Environmental Health Sciences, University of Michigan School of Public Health, Ann Arbor, MI, US
[2]Department of Biostatistics, University of Michigan School of Public Health, Ann Arbor, MI, US
[3]Epidemiology Branch, National Institute of Environmental Health Sciences, Research Triangle Park, NC, US
[4]Division of Maternal and Fetal Medicine, Brigham and Women's Hospital, Harvard Medical School, Boston, MA, US
[5]Department of Internal Medicine-Nephrology, University of Michigan, Ann Arbor, MI, US
[6]Michigan Regional Comprehensive Metabolomics Resource Core, University of Michigan, Ann Arbor, MI, US
[7]Department of Molecular and Integrative Physiology, University of Michigan, Ann Arbor, MI, US
[8]Department of Epidemiology, University of Michigan School of Public Health, Ann Arbor, MI, US

| Plasma Lipid Parent Compounds | |
|---|---|
| α-linolenic acid | [αLA] |
| Arachidonic acid | [AA] |
| Docosahexaenoic acid | [DHA] |
| Eicosapentaenoic acid | [EPA] |
| Linoleic acid | [LA] |

| Plasma Eicosanoids: Cyclooxygenase Pathway | |
|---|---|
| 13,14-dihydro-15-keto-prostaglandin D2 | [3,14DHK-PGD2] |
| 13,14-dihydro-15-keto-prostaglandin F2α | [13,14DHK-PGF2α] |
| 15-deoxy-12,14-prostaglandin J2 | [15DO12,14-PGJ2] |
| 9-oxooctadeca-dienoic acid | [9-oxoODE] |
| Bicyclo-prostaglandin E1 | [BCPGE1] |
| Bicycle-prostaglandin E2 | [BCPGE2] |
| Prostaglandin A2 | [PGA2] |
| Prostaglandin B2 | [PGB2] |
| Prostaglandin D2 | [PGD2] |
| Prostaglandin D3 | [PGD3] |
| Prostaglandin E1 | [PGE1] |
| Prostaglandin E2 | [PGE2] |
| Prostaglandin E3 | [PGE3] |
| Prostaglandin J2 | [PGJ2] |
| Thromboxane B2 | [TXB2] |

| Plasma Eicosanoids: Lipoxygenase Pathway | |
|---|---|
| Resolvin D1 | [RVD1] |
| Resolvin D2 | [RVD2] |
| Leukotriene B4 | [LTB4] |
| Leukotriene C4 methyl-ester | [LTC4-ME] |
| Leukotriene D4 | [LTD4 |
| Leukotriene E4 | [LTE4] |
| 13S-hydroxy-octadecadienoic acid | [13S-HODE] |
| 5-hydroxy-eicosatetraenoic acid | [5-HETE] |
| 8-hydroxy-eicosatetraenoic acid | [8-HETE] |
| 12-hydroxy-eicosatetraenoic acid | [12-HETE] |
| 15-hydroxy-eicosatetraenoic acid | [15-HETE] |
| 13-oxooctadeca-dienoic acid | [13-oxoODE] |
| 5-oxoeicosatetraenoic acid | [5-oxoETE] |
| 12-oxoeicosatetraenoic acid | [12-oxoETE] |
| 15-oxoeicosatetraenoic acid | [15-oxoETE] |

| Plasma Eicosanoids: Cytochrome P450 Pathway | |
|---|---|
| 12,13-dihydroxy-octadecenoic acid | [12,13-DiHOME] |
| 9,10-dihydroxy-octadecenoic acid | [9,10-DiHOME] |
| 5,6-dihydroxy-eicosatrienoic acid | [5,6-DHET] |
| 8,9-dihydroxy-eicosatrienoic acid | [8,9-DHET] |
| 11,12-dihydroxy-eicosatrienoic acid | [11,12-DHET] |
| 5,6-epoxy-eicosatrienoic acid | [5(6)-EET] |
| 8,9-epoxy-eicosatrienoic acid | [8(9)-EET] |
| 14,15-epoxy-eicosatrienoic acid | [14(15)-EET] |
| 11,12-epoxy-eicosatrienoic acid | [11(12)-EET] |
| 9,10-epoxy-octadecenoic acid | [9(10)-EpoME] |
| 12,13-epoxy-octadecenoic acid | [12(13)-EpoME] |
| 20-carboxy arachidonic acid | [CAA] |
| 11-hydroxy-eicosatetraenoic acid | [11-HETE] |
| 16-hydroxy-eicosatetraenoic acid | [16-HETE] |
| 17-hydroxy-eicosatetraenoic acid | [17-HETE] |
| 18-hydroxy-eicosatetraenoic acid | [18-HETE] |
| 20-hydroxy-eicosatetraenoic acid | [20-HETE] |
| 9S-hydroxy-octadecadienoic acid | [9S-HODE] |

| Plasma Inflammation Markers | |
|---|---|
| C-reactive protein | [CRP] |
| Interleukin-1β | [IL1β] |
| Interleukin-6 | [IL6] |
| Interleukin-10 | [IL10] |
| Tumor necrosis factor α | [TNF] |

| Plasma Protein Damage Markers | |
|---|---|
| 3-nitrotyrosine | [NY] |
| *o,o'*-dityrosine | [DY] |
| 3-chlorotyrosine | [CY] |

| Plasma Angiogenic Markers | |
|---|---|
| Placental growth factor | [PGF] |
| Soluble fms-like tyrosine kinase-1 | [sFlt-1] |

| Urinary Oxidative Stress Markers | |
|---|---|
| 8-hydroxydeoxyguanosine | [8-OHdG] |
| 8-isoprostane | [8-IP] |

**Supplemental Figure 1.** Schematic representation of individual biomarkers according to the media in which they were measured and the group they belong to.

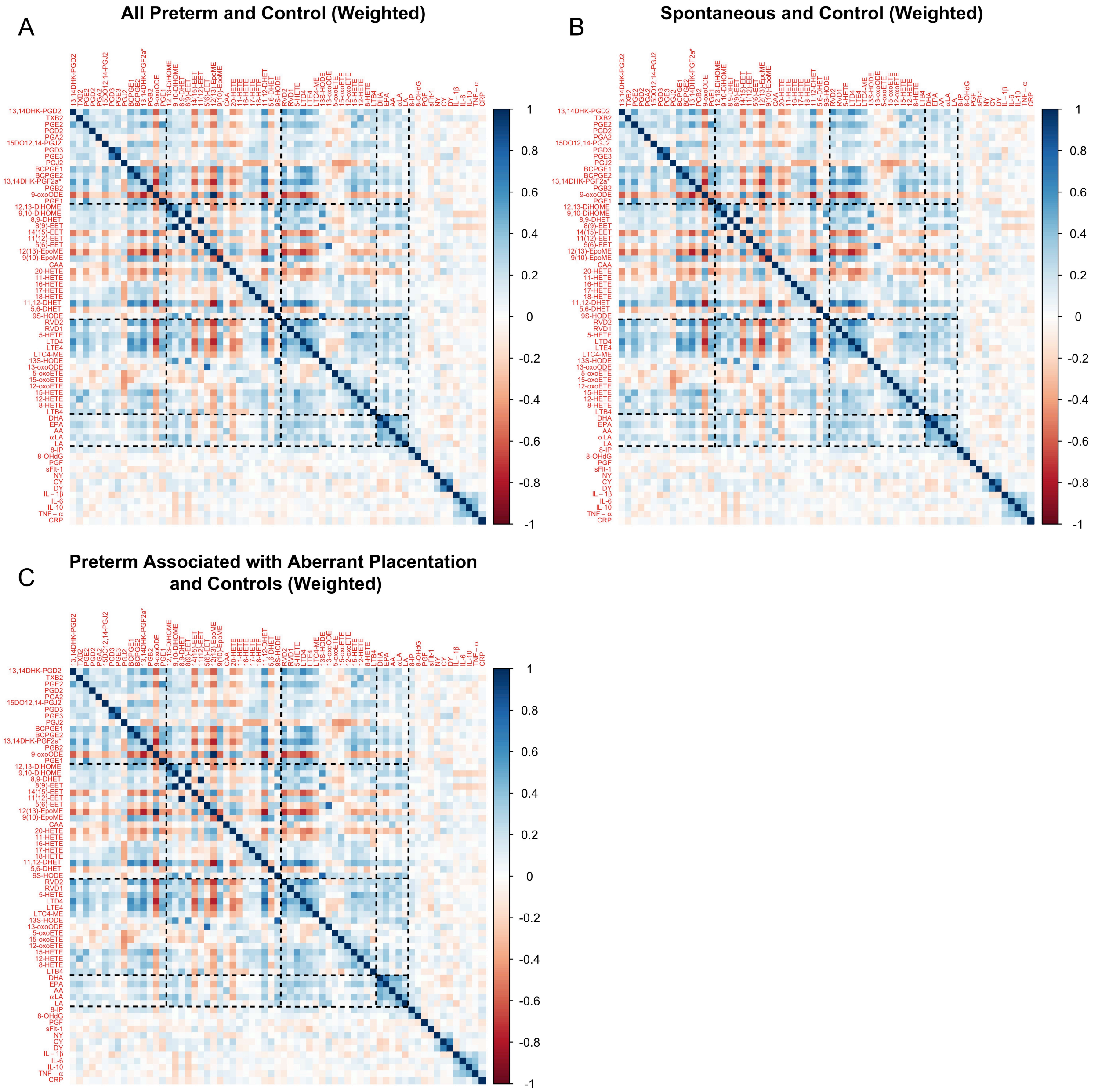


**Supplemental Figure 2. Weighted correlations among biomarkers.** Correlations were calculated using inverse probability weighting for all preterm (A), spontaneous preterm (B), and placental preterm (C) to account for case-control sampling.

**Supplemental Table 1. Log odds ratios (95% confidence interval) of preterm birth estimated using logistic regression and adaptive elastic-net**

| | All Preterm Birth | | Spontaneous Preterm Birth | | Placental Preterm Birth | |
|---|---|---|---|---|---|---|
| Biomarkers | Logistic Regression* | Adaptive Elastic-Net† | Logistic Regression* | Adaptive Elastic-Net† | Logistic Regression* | Adaptive Elastic-Net† |
| **CRP** | -0.18 (-0.54, 0.18) | NA | -0.25 (-0.69, 0.19) | NA | -0.02 (-0.57, 0.53) | NA |
| **TNF-α** | 0.14 (-0.20, 0.48) | NA | -0.06 (-0.46, 0.34) | NA | 0.42 (-0.10, 0.94) | NA |
| **IL-10** | 0.44 (0.09, 0.79) | NA | 0.20 (-0.20, 0.60) | NA | 0.71 (0.22, 1.20) | NA |
| **IL-6** | 0.36 (-0.001, 0.72) | NA | 0.32 (-0.12, 0.75) | NA | 0.26 (-0.25, 0.76) | NA |
| **IL-1β** | 0.14 (-0.21, 0.48) | NA | 0.15 (-0.27, 0.58) | NA | 0.19 (-0.34, 0.71) | NA |
| **DY** | -0.07 (-0.42, 0.27) | NA | -0.01 (-0.45, 0.42) | NA | 0.02 (-0.48, 0.52) | NA |
| **CY** | 0.15 (-0.19, 0.50) | NA | 0.21 (-0.22, 0.64) | NA | 0.15 (-0.36, 0.67) | NA |
| **NY** | 0.26 (-0.12, 0.64) | NA | 0.20 (-0.27, 0.67) | NA | 0.07 (-0.44, 0.58) | NA |
| **sFlt-1** | 0.17 (-0.18, 0.52) | NA | 0.18 (-0.26, 0.62) | NA | 0.05 (-0.46, 0.55) | NA |
| **PGF** | -0.52 (-0.90, -0.14) | -0.24 | -0.28 (-0.73, 0.16) | NA | -1.23 (-1.92, -0.54) | -0.15 |
| **8-OHdG** | -0.23 (-0.60, 0.13) | NA | -0.48 (-0.96, 0.002) | NA | -0.11 (-0.59, 0.36) | NA |
| **8-isoprostane** | 0.02 (-0.40, 0.43) | NA | 0.47 (-0.27, 1.22) | NA | -0.25 (-0.78, 0.28) | NA |
| **13,14DHK-PGD2** | 0.54 (0.14, 0.94) | NA | 1.01 (0.49, 1.53) | NA | -0.15 (-0.63, 0.33) | NA |
| **TXB2** | 0.16 (-0.18, 0.50) | NA | 0.13 (-0.29, 0.54) | NA | 0.18 (-0.32, 0.68) | NA |
| **PGE2** | 0.28 (-0.09, 0.64) | NA | 0.56 (0.05, 1.06) | NA | -0.09 (-0.59, 0.41) | NA |
| **PGD2** | 0.21 (-0.14, 0.57) | NA | 0.24 (-0.20, 0.67) | NA | 0.22 (-0.31, 0.74) | NA |
| **PGA2** | 0.25 (-0.10, 0.61) | NA | 0.41 (-0.05, 0.87) | NA | 0.12 (-0.37, 0.61) | NA |
| **15DO12,14-PGJ2** | 0.41 (0.06, 0.77) | 0.23 | 0.76 (0.27, 1.26) | 0.11 | -0.12 (-0.60, 0.36) | NA |
| **PGD3** | 0.19 (-0.17, 0.55) | NA | 0.42 (-0.04, 0.89) | NA | -0.04 (-0.54, 0.46) | NA |
| **PGE3** | 0.01 (-0.34, 0.37) | NA | 0.07 (-0.38, 0.52) | NA | -0.02 (-0.53, 0.50) | NA |
| **PGJ2** | -0.15 (-0.51, 0.20) | NA | -0.48 (-0.93, -0.02) | NA | 0.23 (-0.37, 0.83) | NA |
| **BCPGE1** | 0 (-0.34, 0.34) | NA | 0.23 (-0.18, 0.64) | NA | -0.62 (-1.23, -0.01) | NA |
| **BCPGE2** | 0.06 (-0.28, 0.40) | NA | 0.23 (-0.18, 0.65) | NA | -0.55 (-1.12, 0.03) | NA |
| **13,14DHK-PGF2a*** | 0.19 (-0.15, 0.54) | NA | 0.61 (0.18, 1.05) | NA | -0.49 (-1.07, 0.09) | NA |
| **PGB2** | 0.30 (-0.13, 0.72) | NA | 0.65 (0.07, 1.23) | NA | -0.10 (-0.56, 0.36) | NA |
| **9-oxoODE** | -0.36 (-0.70, -0.02) | NA | -0.65 (-1.06, -0.25) | NA | 0.30 (-0.33, 0.93) | NA |
| **PGE1** | 0.16 (-0.19, 0.51) | NA | 0.34 (-0.11, 0.79) | NA | -0.23 (-0.71, 0.26) | NA |
| **12,13-DiHOME** | 0.32 (-0.02, 0.66) | NA | 0.46 (0.05, 0.87) | NA | 0.14 (-0.35, 0.64) | NA |
| **9,10-DiHOME** | 0.37 (-0.01, 0.74) | NA | 0.65 (0.15, 1.16) | NA | -0.06 (-0.58, 0.45) | NA |
| **8,9-DHET** | 0.49 (0.06, 0.91) | NA | 0.78 (0.18, 1.37) | NA | -0.001 (-0.51, 0.51) | NA |
| **8(9)-EET** | 0.37 (-0.01, 0.74) | NA | 0.65 (0.15, 1.16) | NA | -0.06 (-0.58, 0.45) | NA |
| **14(15)-EET** | -0.09 (-0.43, 0.25) | NA | -0.26 (-0.65, 0.13) | NA | 0.49 (-0.18, 1.16) | NA |

| | | | | | | |
|---|---|---|---|---|---|---|
| **11(12)-EET** | 0.48 (0.06, 0.90) | NA | 0.77 (0.18, .135) | NA | -0.002 (-0.51, 0.50) | NA |
| **5(6)-EET** | -0.37 (-0.72, -0.01) | -0.10 | -0.63 (-1.08, -0.18) | -0.16 | 0.30 (-0.26, 0.86) | NA |
| **12(13)-EpoME** | -0.36 (-0.70, -0.02) | NA | -0.65 (-1.06, -0.25) | NA | 0.30 (-0.33, 0.93) | NA |
| **9(10)-EpoME** | 0.42 (0.04, 0.79) | NA | 0.91 (0.37, 1.46) | NA | -0.20 (-0.70, 0.31) | NA |
| **CAA** | 0.09 (-0.25, 0.43) | NA | 0.25 (-0.19, 0.69) | NA | -0.09 (-0.57, 0.39) | NA |
| **20-HETE** | -0.13 (-0.47, 0.22) | NA | -0.35 (-0.77, 0.07) | NA | 0.49 (-0.13, 1.11) | NA |
| **11-HETE** | -0.07 (-0.41, 0.27) | NA | -0.36 (-0.78, 0.05) | NA | 0.52 (-0.06, 1.10) | NA |
| **16-HETE** | 0.04 (-0.31, 0.39) | NA | 0.10 (-0.35, 0.55) | NA | 0.01 (-0.50, 0.51) | NA |
| **17-HETE** | -0.02 (-0.36, 0.32) | NA | 0.19 (-0.24, 0.62) | NA | -0.29 (-0.81, 0.23) | NA |
| **18-HETE** | -0.002 (-0.34, 0.34) | NA | 0.33 (-0.11, 0.76) | NA | -0.30 (-0.82, 0.23) | NA |
| **11,12-DHET** | 0.5 (0.15, 0.86) | NA | 0.96 (0.52, 1.39) | NA | -0.78 (-1.58, 0.03) | NA |
| **5,6-DHET** | -0.10 (-0.44, 0.25) | NA | -0.31 (-0.74, 0.12) | NA | 0.29 (-0.24, 0.81) | NA |
| **9S-HODE** | 0.14 (-0.20, 0.48) | NA | 0.27 (-0.16, 0.70) | NA | 0.03 (-0.48, 0.55) | NA |
| **RVD2** | 0.21 (-0.12, 0.55) | NA | 0.46 (0.05, 0.87) | NA | -0.39 (-0.96, 0.17) | NA |
| **RVD1** | 0.65 (0.25, 1.05) | NA | 0.98 (0.47, 1.49) | 0.02 | 0.14 (-0.44, 0.73) | NA |
| **5-HETE** | 0.34 (-0.002 ,0.69) | NA | 0.69 (0.25, 1.13) | NA | -0.16 (-0.66, 0.34) | NA |
| **LTD4** | 0.38 (0.03, 0.72) | NA | 0.88 (0.44, 1.32) | NA | -0.80 (-1.57, -0.03) | NA |
| **LTE4** | 0.38 (-0.02, 0.89) | NA | 0.67 (0.17, 1.17) | NA | -0.18 (-0.86, 0.50) | NA |
| **LTC4-ME** | 0.30 (-0.07, 0.66) | NA | 0.53 (0.04, 1.03) | NA | -0.10 (-0.56, 0.36) | NA |
| **13S-HODE** | 0.28 (-0.08, 0.64) | NA | 0.45 (-0.01, 0.91) | NA | 0.33 (-0.22, 0.88) | NA |
| **13-oxoODE** | -0.25 (-0.61, 0.10) | NA | -0.39 (-0.83, 0.05) | NA | 0.21 (-0.34, 0.76) | NA |
| **5-oxoETE** | 0.28 (-0.08, 0.64) | NA | 0.57 (0.10, 1.05) | 0.16 | -0.16 (-0.68, 0.37) | NA |
| **15-oxoETE** | 0.17 (-0.19, 0.52) | NA | 0.12 (-0.33, 0.57) | NA | 0.26 (-0.27, 0.79) | NA |
| **12-oxoETE** | 0.27 (-0.09, 0.63) | NA | 0.53 (0.06, 1.00) | NA | -0.20 (-0.72, 0.32) | NA |
| **15-HETE** | 0.39 (0.02, 0.77) | NA | 0.67 (0.19, 1.15) | NA | -0.002 (-0.51, 0.50) | NA |
| **12-HETE** | 0.37 (0.01, 0.74) | NA | 0.51 (0.04, 0.98) | NA | 0.12 (-0.37, 0.62) | NA |
| **8-HETE** | 0.29 (-0.09, 0.66) | NA | 0.49 (-0.04, 1.01) | NA | -0.01 (-0.49, 0.47) | NA |
| **LTB4** | -0.31 (-0.67, 0.04) | -0.13 | 0.05 (-0.37, 0.47) | NA | -0.83 (-1.42, -0.23) | -0.10 |
| **DHA** | 0.36 (0, 0.73) | NA | 0.84 (0.35, 1.34) | NA | -0.44 (-1.04, 0.16) | NA |
| **EPA** | 0.43 (0.06, 0.79) | NA | 0.87 (0.37, 1.37) | NA | -0.29 (-0.84, 0.25) | NA |
| **AA** | 0.29 (-0.06, 0.64) | NA | 0.56 (0.10, 1.02) | NA | -0.08 (-0.59, 0.42) | NA |
| **$\alpha$LA** | 0.50 (0.11, 0.88) | NA | 0.89 (0.33, 1.44) | NA | 0.07 (-0.47, 0.61) | NA |
| **LA** | 0.36 (-0.003, 0.72) | 0.26 | 0.54 (0.07, 1.00) | NA | 0.20 (-0.34, 0.74) | NA |

[*]Log odds ratios were estimated by fitting logistic regression on covariates and individual biomarkers (*Model 1*)
[†]Only point estimates are reported for the log odds ratios estimated by adaptive elastic-net. Zero coefficients are recorded as NA.
